# Evaluating large language models for diabetic retinopathy multiple-choice question generation in clinical ophthalmic education

**DOI:** 10.3389/fmed.2026.1874243

**Published:** 2026-07-15

**Authors:** Xue Qin, Ping Song, Zhipeng Yan, Hui Qian, Ligang Jiang, Chenghu Wang

**Affiliations:** 1 The Affiliated Eye Hospital of Nanjing Medical University, Nanjing, Jiangsu, China; 2 Department of Ophthalmology, Quzhou Affiliated Hospital of Wenzhou Medical University, Quzhou People’s Hospital, Quzhou, Zhejiang, China

**Keywords:** artificial intelligence, benchmarking study, clinical ophthalmic education, diabetic retinopathy, large language models, multiple-choice questions

## Abstract

**Background:**

Large language models (LLMs) are increasingly used in medical education, but their ability to generate ophthalmic multiple-choice questions (MCQs) remains unclear. Diabetic retinopathy (DR), a core ophthalmic training topic, provides a framework for evaluating LLM-based item generation.

**Methods:**

Five publicly accessible LLMs completed 60 predefined DR MCQ tasks under a standardized Chinese single-turn prompt and blueprint, yielding 300 items. Evaluation included structural completeness, format compliance, keyed-answer accuracy, textual features, response time, and blinded expert ratings across six educational domains. Because the same 60 tasks were completed by all five models, between-model comparisons were performed using paired task-level analyses. Continuous and ordinal outcomes were compared using Friedman tests, followed by Bonferroni-corrected paired Wilcoxon signed-rank tests when appropriate. Inter-rater reliability was assessed using intraclass correlation coefficients, and Spearman analyses examined associations between output features and expert-rated quality.

**Results:**

Between-model differences were observed in all textual variables and response time (all Friedman test *p* < 0.001). Gemini 3 was fastest (6.47 ± 1.27 s), whereas Qwen3-Max-Thinking was slowest (21.99 ± 4.38 s). Gemini 3 and ChatGPT-5.4 produced the longest responses (239.67 ± 29.12 and 245.98 ± 39.61 characters, respectively). ICCs ranged from 0.794 to 0.859. Between-model differences were found for content rigor, clarity, distractor quality, cognitive-level alignment, overall usability, and mean score, whereas educational usefulness did not reach statistical significance. For overall usability, the overall Friedman test showed only a weak difference, and no pairwise comparison remained significant after Bonferroni correction. Gemini 3 showed the highest proportion of directly usable items (90.00%), followed by ChatGPT-5.4 (86.67%). Longer explanations were associated with higher expert-rated quality, whereas longer response time showed no quality advantage.

**Conclusion:**

All five LLMs generated structurally complete and format-compliant DR MCQ drafts, but differences remained in factual accuracy, expert-rated item quality, output style, and usability. Gemini 3 and ChatGPT-5.4 showed the most favorable balance between correctness and expert-rated usability, supporting LLMs as assisted item-generation tools rather than replacements for expert review.

## 1 Introduction

In recent years, the rapid development of large language models (LLMs) has substantially expanded the application boundaries of generative artificial intelligence in medical education (1–6). Existing studies have primarily focused on the performance of LLMs in medical examination answering, clinical question answering, case discussion, patient education, and academic writing assistance (7–11). However, their potential value on the “generation side” of educational content, particularly in test-item construction, remains insufficiently evaluated (12). In medical subspecialties, where item banks require frequent updating and faculty often face a substantial assessment-design burden, LLMs could become an important tool for human–machine collaborative education if they are able to generate questions that are consistently accurate in content, structurally standardized, and educationally valuable.

Single-best-answer multiple-choice questions (MCQs) are among the most commonly used assessment formats in medical education and are widely applied in undergraduate teaching, standardized residency training, subspecialty training, and both formative and summative assessment (13). High-quality MCQs can assess not only learners’ mastery of foundational knowledge, but also their clinical reasoning, differential diagnosis, and decision-making abilities. Compared with general text generation, however, medical MCQ construction places substantially higher demands on content accuracy, stem clarity, distractor quality, the presence of a single best answer, and cognitive-level alignment. Deficiencies in item construction may compromise test validity and affect fairness in candidate performance. Accordingly, the suitability of LLMs for medical question generation cannot be judged solely on the basis of linguistic fluency, but instead requires systematic evaluation from both educational and disciplinary perspectives.

Ophthalmic education is highly specialized and clinically integrative. Its teaching content encompasses not only foundational knowledge such as anatomy, physiology, and pathology, but also multi-level competency training in symptom recognition, disease staging, interpretation of ancillary examinations, treatment decision-making, and long-term follow-up management. Diabetic retinopathy (DR), one of the most common and clinically important ocular complications of diabetes, is also a major cause of preventable visual impairment among working-age adults worldwide (14–16). Its educational content spans key domains including disease definition, staging logic, typical fundus signs, diabetic macular edema (DME), screening and follow-up principles, systemic risk-factor management, and treatment-strategy selection (17). DR therefore represents the continuum from foundational knowledge to clinical decision-making in ophthalmic education and provides an ideal entry point for evaluating the subspecialty item-generation capabilities of LLMs (18). In addition, DR is one of the most active and representative disease areas in contemporary ophthalmic artificial intelligence research, which further supports its suitability as a disease scenario for evaluating LLM-based subspecialty question generation (19, 20).

In ophthalmology, only a limited number of recent studies have begun to compare the quality of ophthalmic educational MCQs generated by LLMs. Although these early findings suggest potential utility, more systematic validation with a tighter disease-specific focus is still needed (21). In particular, it remains unclear whether major publicly accessible LLMs differ consistently in content accuracy, item-writing quality, distractor quality, educational usefulness, and cognitive-level alignment when generating DR educational MCQs. Against this background, the present study used DR as a model disease topic, developed a standardized item-generation blueprint and a unified Chinese prompt template, and compared the performance of five publicly accessible LLMs in generating DR educational single-best-answer MCQs. Across four content domains—foundational knowledge, clinical cases, treatment decision-making, and screening/follow-up management—we combined objective evaluation metrics with blinded expert scoring to systematically assess the structural completeness, correct answer accuracy, item-writing quality, distractor quality, content rigor, educational usefulness, and overall usability of model-generated questions. We hope that this study will provide empirical evidence for the use of LLMs in item generation for ophthalmic subspecialty education and inform the future development of high-quality human–machine collaborative workflows for question-bank generation and review.

## 2 Materials and methods

### 2.1 Study design

**Figure 1:**
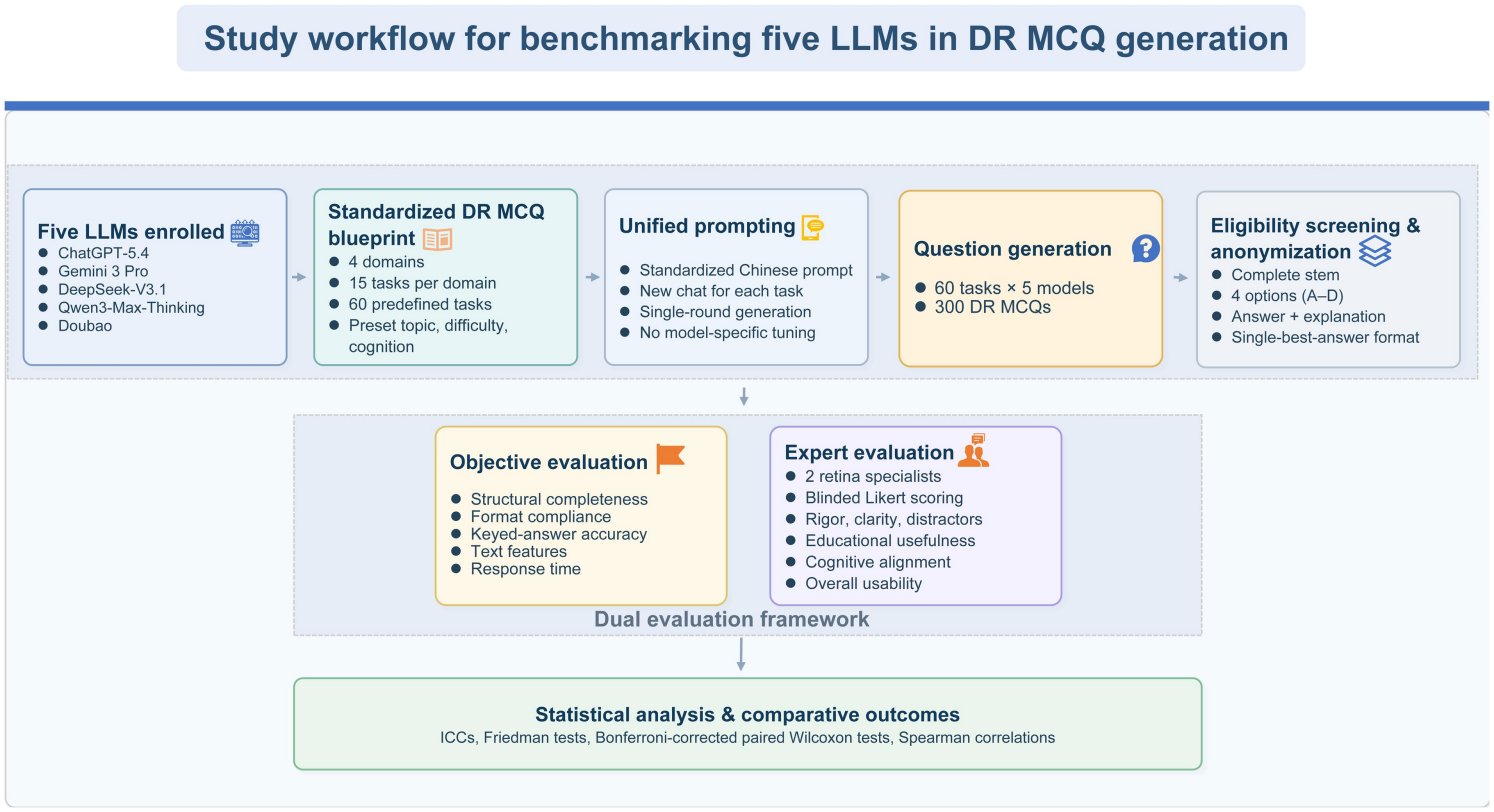
Study workflow for benchmarking five publicly accessible large language models (LLMs) in diabetic retinopathy (DR) multiple-choice question (MCQ) generation. The study used a standardized DR MCQ blueprint with four content domains and 60 prespecified tasks. A unified Chinese prompt and single-turn generation workflow were applied to five LLMs, producing 300 DR MCQs. After eligibility screening and anonymization, model outputs were evaluated using a dual framework consisting of objective assessment and blinded expert scoring by two retinal specialists. Final analyses included inter-rater agreement, between-model comparisons, and exploratory correlation analysis.

This comparative benchmarking study was conducted in a Chinese-prompt and Chinese-output setting. The study was designed to systematically evaluate performance differences among five LLMs in generating DR educational single-best-answer MCQs. The primary focus was the models’ item-generation capability and educational applicability, rather than their performance in answering pre-existing examination questions. To enhance comparability across models, we prespecified a unified DR item-generation blueprint and applied a standardized Chinese prompt template, a uniform generation workflow, and a multidimensional evaluation framework to systematically assess model-generated content (Figure 1).

### 2.2 Target models and access conditions

Five publicly accessible LLM-based chat systems were included in this study. For each platform, evaluation was conducted using the high-performance general-purpose or reasoning mode that was visibly available to ordinary users and could be directly selected through the platform’s official public web interface during the testing period. All testing was performed between March 6 and March 10, 2026. To improve reproducibility, we recorded the model names, mode labels, version identifiers, and other relevant visible settings displayed in the user interface of each platform during testing. Specifically, the ChatGPT platform was tested using GPT-5.4 Thinking mode; the Gemini platform using Gemini 3 Thinking mode; the DeepSeek platform using DeepSeek-V3.1 Deep Thinking mode; the Qwen platform using Qwen3-Max Thinking mode; and the Doubao platform using Thinking mode.

Because public web interfaces differ in the extent to which underlying generation parameters are exposed, we did not attempt to standardize non-visible parameters across platforms. Instead, we standardized only user-controllable conditions, including access through official web interfaces, use of a uniform user prompt template, completion of each task in a newly initiated independent chat session, and adherence to a consistent task workflow. For platforms offering enhanced functions such as web search, code interpreter, or other external tools, these additional capabilities were not proactively enabled during formal testing. We also did not apply model-specific prompt optimization, add open-ended follow-up prompts, or conduct iterative multi-turn negotiation-style rewriting. All original chat records and outputs were fully archived for subsequent data extraction, verification, and review.

### 2.3 Development of the DR single-best-answer MCQ blueprint

Before formal testing, we developed a standardized blueprint for DR single-best-answer MCQs to unify the scope, knowledge structure, and cognitive levels of item generation. The item content was restricted to DR education and divided into four prespecified content domains: (1) foundational knowledge; (2) clinical cases; (3) treatment decision-making; and (4) screening/follow-up management. The four domains were selected to cover the main continuum of DR education, from basic disease concepts and lesion recognition to clinical scenario interpretation, treatment selection, and longitudinal screening and follow-up.

Each content domain comprised 15 task units, yielding a total of 60 standardized item-generation tasks. For each task unit, the content category, specific knowledge point, difficulty level, and cognitive level were prespecified. Difficulty was classified as easy, moderate, or difficult, and cognitive level as recall, understanding, application, or analysis. For example, foundational-knowledge tasks covered the definition and staging logic of DR, typical fundus signs, and diabetic macular edema; clinical-case tasks required interpretation of symptom and examination descriptions; treatment-decision tasks addressed anti-VEGF therapy, laser photocoagulation, vitrectomy-related indications, and systemic risk-factor management; and screening/follow-up tasks covered examination intervals, referral indications, and patient-management principles. The complete 60-task blueprint, including domain, knowledge point, difficulty level, and cognitive level for each task, is provided in Supplementary material 1.

Because no mature sample-size calculation method is currently available for this type of benchmarking study, the number of tasks was determined pragmatically to balance content coverage, representativeness across domains, and the feasibility of blinded expert scoring. The 15 tasks within each domain were distributed to avoid overrepresentation of any single knowledge point, and the same 60 blueprint items were directly incorporated into the standardized prompt template for all five models. Thus, each model generated items for exactly the same educational tasks, with only the model identity differing across runs. This blueprint ensured fairness of cross-model comparisons and reduced bias arising from uneven knowledge-point distribution or differences in item structure.

### 2.4 Prompt design and item-generation workflow

A unified Chinese prompt template was designed and used for all models in this study. The prompt instructed each model to generate one DR single-best-answer MCQ for ophthalmology residents in standardized residency training and explicitly specified the topic, content category, specific knowledge point, difficulty level, cognitive level, output format, and item-writing constraints. The full standardized prompt template in both Chinese and English, together with the standardized remedial prompt, is provided in Supplementary material 2.

The unified prompt required each item to include a stem, four options (A–D), a single best answer, and a brief explanation. It further specified that each question must conform to the single-best-answer MCQ format, with only one uniquely correct answer. Incorrect options were required to have plausible distractor quality, but could not be obviously implausible, semantically duplicative of the correct answer, or equally valid under common clinical scenarios. The prompt also required the stem to be clearly worded, logically coherent, and sufficiently informative, while avoiding double negatives, excessive cueing, and dependence on real images. If examination findings were involved, they could be presented only in the form of textual descriptions. The 60 task units were separately entered into the five target models, with only one item generated per interaction, yielding a total of 300 model-generated MCQs. All items were generated in a single-turn manner and completed in the prespecified order.

If the initial output did not meet the prespecified format requirements, one standardized remedial prompt was allowed for regeneration. If the regenerated output still failed to meet the requirements, it was recorded as a generation failure, retained in the format analysis, and excluded from subsequent content-quality scoring.

### 2.5 Inclusion criteria and output processing

Items were eligible for analysis only if they met all of the following criteria: a complete stem; four options labeled A–D; a clearly identified correct answer; an explanation; conformity to the single-best-answer MCQ format; and only one uniquely correct answer. Outputs were judged structurally ineligible if they lacked any required field, contained more than one defensible correct answer, used prohibited options such as “All of the above” or “None of the above,” or required an unavailable real image for the question to be answered.

All eligible items were anonymized, pooled across the five models and four domains, and presented in a fully randomized order before expert scoring to minimize rater bias as much as possible. Before formal evaluation, no manual rewriting, factual correction, or language polishing was performed on model outputs; apart from necessary formatting arrangement, their original output characteristics were preserved to the greatest extent possible. Items that failed to meet the structural inclusion criteria were excluded from subjective quality scoring, but were retained for analysis of the generation failure rate and other related objective evaluations.

### 2.6 Reference key and evaluation manual

Before formal evaluation, a standardized reference standard was developed to support consistent judgment of correct-answer accuracy, content rigor, and explanation consistency. The reference standard was prepared by the research team before model-output evaluation, based on mainstream ophthalmology textbooks, DR-related guidelines or consensus statements, and standardized ophthalmology residency-training materials (22–24). It covered the core knowledge areas represented in the prespecified DR MCQ blueprint, including the definition of DR, non-proliferative and proliferative DR, diabetic macular edema, typical fundus manifestations, systemic risk factors, screening and follow-up principles, and major treatment strategies.

The initial reference standard was drafted by retina-trained ophthalmologists and reviewed within the research team to ensure consistency with mainstream ophthalmic education and current clinical practice. When differences existed among reference sources, priority was given to the most recent DR-related guidelines or consensus statements and to recommendations consistent with mainstream ophthalmic education. The reference standard was finalized before any model outputs were reviewed and was checked by a senior retinal specialist who was not involved in the initial drafting process. For each blueprint task, the reference standard specified the expected knowledge focus, acceptable clinical reasoning, key points required for a medically correct answer, and common inaccurate or misleading interpretations. During evaluation, it was used to determine whether the model-indicated answer was medically correct, whether only one best answer was defensible, whether the explanation was consistent with the correct answer, and whether the generated item was appropriate for DR education.

In parallel, a prespecified evaluation manual was developed to standardize objective evaluation, expert Likert-scale scoring, categorical usability conversion, and discrepancy-resolution procedures. The reference standard and evaluation manual are provided in Supplementary material 3.

### 2.7 Objective evaluation metrics

A prespecified objective evaluation framework was used to systematically assess the items generated by each model, mainly covering the following aspects:

(1) Structural completeness: whether the model output contained all essential components, including a stem, four options, the correct answer, and an explanation. The complete output rate was calculated for each model.
(2) Format compliance: whether the model output conformed to the prespecified single-best-answer MCQ format, including provision of four options labeled A–D, clear identification of a single correct answer, and absence of multiple-answer structures or prohibited option formats.
(3) Correct answer accuracy: whether the answer identified by the model was truly correct. Judgments were based on the prespecified reference standard. Outputs were classified as inaccurate if the indicated answer was incorrect, ambiguous, inconsistent with the explanation, or unsupported by a reasonable medical rationale.
(4) Textual output characteristics: because Chinese word-segmentation methods may affect the stability of word-count statistics, character count was used as the primary measure of text length. The recorded indicators included stem length, explanation length, total response length, mean option length, and option length SD. Option length SD was defined as the standard deviation of the character counts across the four options and was used to reflect the extent of imbalance in option length.
(5) Generation efficiency: assessed by response time and recorded by manual timing. Timing started when the prompt was submitted and ended when the model’s visible output was completed in the chat interface. This metric reflects the user-perceived generation efficiency under public web-interface conditions.

### 2.8 Expert subjective evaluation

Subjective quality evaluation was independently conducted by two ophthalmology experts with subspecialty training in retina and experience in medical education. Both raters were retina-trained ophthalmologists with experience in DR diagnosis and management, ophthalmology residency teaching, and MCQ-based educational assessment. Their years of clinical practice, teaching experience, and prior involvement in MCQ construction were recorded before formal scoring. Before formal scoring, eight pilot items that were not included in the final analysis were used for calibration. These pilot items were generated separately from the formal 60-task blueprint and were used only to harmonize the interpretation of scoring anchors, single-best-answer judgments, and usability categories. All items included for scoring were evaluated under anonymized and blinded conditions.

Each item was rated on a 5-point Likert scale across the following six domains:

(1) Content rigor: whether the item content, option design, and explanation were consistent with mainstream ophthalmic education and the clinical knowledge framework related to DR;
(2) Clarity: whether the stem and options were clearly expressed, logically presented, and easy to understand;
(3) Distractor quality: whether the incorrect options had reasonable plausibility and educational value;
(4) Educational usefulness: whether the item was suitable for ophthalmic teaching or formative assessment;
(5) Cognitive-level alignment: whether the generated item matched the prespecified cognitive level in the task blueprint;
(6) Overall usability: the expert’s global judgment of whether the item could be used directly, used after minor revision, or was not recommended for educational use without major revision. This domain was scored on a 5-point Likert scale, with 5 indicating direct usability, 4 indicating usability after minor revision, and scores of 1–3 indicating that the item was not recommended for use without major revision.

For ease of result presentation and educational interpretation, the 5-point overall usability score was prespecified to be collapsed into three categories: directly usable (score = 5), usable after minor revision (score = 4), and not recommended (score = 1–3). These categories were therefore directly derived from the Likert-scale overall usability score rather than assessed as an independent measure.

### 2.9 Inter-rater agreement and discrepancy resolution

To evaluate agreement between the two experts’ subjective ratings, inter-rater agreement metrics were calculated. For Likert-scale ratings, the intraclass correlation coefficient (ICC) was used to assess agreement, and 95% CIs were reported. The independent Likert-scale ratings were retained for inter-rater reliability analysis. For key judgments requiring categorical or final item-level decisions, including correct-answer accuracy, structural eligibility assessment, explanation consistency, and overall usability classification, any disagreement between the two experts was rechecked against the reference standard and evaluation manual. Disagreements were first resolved through discussion; if consensus could not be reached, a third senior retinal specialist was consulted for adjudication. The three-category overall usability classification was derived from the final overall usability score according to the prespecified conversion rule.

### 2.10 Statistical analysis

Statistical analyses were performed using IBM SPSS Statistics for Windows, version 27.0 (IBM Corp., Armonk, NY, USA). Categorical variables are presented as counts and percentages, whereas continuous variables are presented as mean ± standard deviation or median (interquartile range), as appropriate according to their distribution. Because the same 60 prespecified blueprint tasks were completed by all five models, between-model comparisons were performed using paired task-level analyses. Continuous and ordinal outcomes were compared using Friedman tests, with task ID treated as the blocking factor. When the overall Friedman test was statistically significant, *post hoc* pairwise comparisons were performed using paired Wilcoxon signed-rank tests with Bonferroni correction. Kendall’s W was reported as the effect-size measure for Friedman tests. Binary or categorical objective outcomes were summarized descriptively as *n* (%), and Wilson 95% confidence intervals were reported for key proportions, including correct-answer accuracy and directly usable classification. Statistical comparisons were not performed for indicators showing no between-model variability. Inter-rater agreement for expert ratings was assessed using intraclass correlation coefficients, with 95% confidence intervals reported. Spearman correlation analysis was used to explore the relationships among textual output characteristics, generation efficiency, and expert subjective ratings, and the Spearman correlation coefficient (*ρ*) and corresponding *p* value were reported. All tests were two-sided, and *p* < 0.05 was considered statistically significant.

## 3 Results

### 3.1 Overall generation outcomes and objective evaluation results

All five LLMs completed the prespecified 60 DR single-best-answer MCQ generation tasks, yielding a total of 300 items. For all models, the initial outputs met the structural eligibility criteria, and all final outputs were structurally complete and compliant with the prespecified format requirements. Accordingly, all items were included in the blinded subjective evaluation process. During formal testing, no model required the standardized remedial prompt, and no generation failures occurred. The relevant objective evaluation results are presented in Table 1.

**Table 1:** Objective evaluation results for DR educational MCQs generated by five LLMs.

Indicator	ChatGPT-5.4 (*n* = 60)	Gemini 3 (*n* = 60)	DeepSeek-V3.1 (*n* = 60)	Qwen3-Max-Thinking (*n* = 60)	Doubao (*n* = 60)
Structurally qualified on first output, *n* (%)	60 (100.00)	60 (100.00)	60 (100.00)	60 (100.00)	60 (100.00)
Qualified after one standardized remedial prompt, *n* (%)	0 (0.00)	0 (0.00)	0 (0.00)	0 (0.00)	0 (0.00)
Final structurally complete output, *n* (%)	60 (100.00)	60 (100.00)	60 (100.00)	60 (100.00)	60 (100.00)
Format compliant, *n* (%)	60 (100.00)	60 (100.00)	60 (100.00)	60 (100.00)	60 (100.00)
Correct answer accurate, *n* (%) [95% CI]	60 (100.00) [93.98, 100.00]	60 (100.00) [93.98, 100.00]	58 (96.67) [88.64, 99.08]	55 (91.67) [81.93, 96.39]	55 (91.67) [81.93, 96.39]
Included in subjective rating, *n* (%)	60 (100.00)	60 (100.00)	60 (100.00)	60 (100.00)	60 (100.00)
Generation failure, *n* (%)	0 (0.00)	0 (0.00)	0 (0.00)	0 (0.00)	0 (0.00)

Data are presented as *n* (%), with percentages calculated using 60 prespecified generation tasks per model as the denominator. Wilson 95% CIs are provided for correct-answer accuracy because it represents a key objective proportion. “Structurally qualified on first output” indicates that the initial response met all prespecified structural inclusion criteria. “Qualified after one standardized remedial prompt” indicates outputs that became structurally eligible after one rescue prompt. “Final structurally complete output” indicates that the output contained a question stem, 4 A–D options, an explicitly identified correct answer, and an explanation. “Format compliant” refers to conformity with the prespecified single-best-answer multiple-choice question format. “Correct answer accurate” was judged against the prespecified reference standard. “Included in subjective rating” refers to structurally eligible outputs that entered blinded expert scoring. “Generation failure” refers to outputs that remained structurally non-compliant after the allowed remedial step. Statistical comparisons were not performed for indicators showing no between-model variability.

CI, confidence interval; DR, diabetic retinopathy; LLM, large language model; MCQ, multiple-choice question.

As shown in Table 1, all five models achieved 60/60 (100.00%) for structurally qualified on first output, final structurally complete output, format compliance, and inclusion in subjective rating. Because structural completeness, format compliance, inclusion in subjective rating, and generation failure showed no between-model variability, statistical comparisons were not applicable for these indicators. Correct-answer accuracy was 60/60 (100.00%; 95% CI: 93.98–100.00%) for ChatGPT-5.4 and Gemini 3, 58/60 (96.67%; 95% CI: 88.64–99.08%) for DeepSeek-V3.1, and 55/60 (91.67%; 95% CI: 81.93–96.39%) for both Qwen3-Max-Thinking and Doubao (Table 1). These findings indicate that, under the unified prompt framework, all models were able to stably generate structurally eligible DR educational single-best-answer MCQs, although some differences in content accuracy remained.

### 3.2 Textual output characteristics and generation efficiency

Using paired task-level analyses, significant differences were observed among the models for all textual output characteristics and generation efficiency metrics (all Friedman test *p* < 0.001), as shown in Table 2.

**Table 2:** Comparison of textual output characteristics and generation efficiency across five LLMs.

Indicator	ChatGPT-5.4 (*n* = 60)	Gemini 3 (*n* = 60)	DeepSeek-V3.1 (*n* = 60)	Qwen3-max-thinking (*n* = 60)	Doubao (*n* = 60)	Friedman χ^2^	Kendall’s W	*p*-Values
Stem length	51.50 (29.00, 79.00)	36.00 (31.00, 56.75)	50.50 (24.25, 80.00)	30.50 (23.25, 40.75)	27.00 (23.00, 35.75)	96.880	0.404	**<0.001**
Mean option length	16.89 ± 4.41	14.62 ± 5.19	10.25 (7.56, 12.69)	9.00 (5.25, 10.25)	12.43 ± 3.86	121.262	0.505	**<0.001**
Explanation length	128.00 (110.25,137.00)	134.03 ± 11.93	59.50 (48.00, 73.00)	75.58 ± 14.52	81.07 ± 4.24	184.678	0.769	**<0.001**
Total response length	245.98 ± 39.61	239.67 ± 29.12	153.50 (129.00,183.75)	143.00 (126.00,160.25)	162.80 ± 18.35	174.370	0.727	**<0.001**
Option length SD	3.44 (2.33, 5.82)	4.23 (2.41, 5.85)	3.90 (1.57, 4.82)	1.54 (1.30, 2.54)	4.23 ± 2.20	40.830	0.170	**<0.001**
Response time	9.54 ± 1.43	6.47 ± 1.27	16.58 ± 3.46	21.99 ± 4.38	9.45 ± 1.47	207.152	0.863	**<0.001**

Text-length variables are expressed as character counts. Option length SD refers to the standard deviation of the lengths of the four answer options. Response times are expressed in seconds. Values are presented as mean ± SD or median (Q1, Q3), as appropriate according to the descriptive distribution of each model-specific metric. Overall between-model comparisons were performed using the Friedman test, with task ID treated as the blocking factor. Kendall’s W was reported as the effect-size measure for the Friedman test. When the overall Friedman test was statistically significant, post hoc pairwise comparisons were performed using paired Wilcoxon signed-rank tests with Bonferroni correction. The *p*-values shown in the table refer to overall Friedman test results. Bold *p*-values indicate statistically significant overall between-model differences (*p* < 0.05).

LLM, large language model; Q1, first quartile; Q3, third quartile; SD, standard deviation.

**Figure 2:**
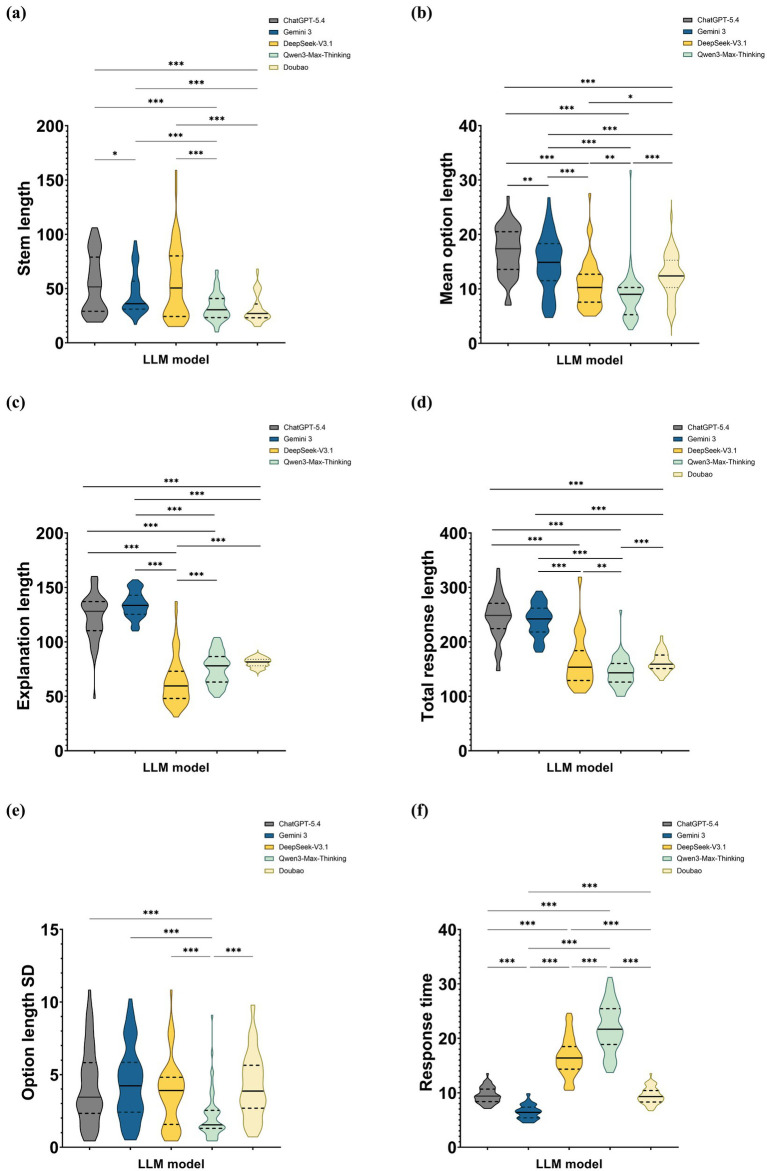
Comparison of textual output characteristics and generation efficiency across five large language models (LLMs). Violin plots show the distributions of **(a)** stem length, **(b)** mean option length, **(c)** explanation length, **(d)** total response length, **(e)** option length standard deviation (SD), and **(f)** response time for diabetic retinopathy (DR) multiple-choice questions (MCQs) generated by the five LLMs. Text-length variables are expressed as character counts, and response time is expressed in seconds. The central solid line indicates the median, and the dashed lines indicate the interquartile range. Brackets denote significant pairwise differences after paired Wilcoxon signed-rank tests with Bonferroni correction. *p < 0.05; **p < 0.01; ***p < 0.001 after Bonferroni correction.

For stem length, ChatGPT-5.4 and DeepSeek-V3.1 generated relatively longer stems, with medians of 51.50 (29.00, 79.00) and 50.50 (24.25, 80.00) characters, respectively. Gemini 3 was intermediate at 36.00 (31.00, 56.75) characters, whereas Qwen3-Max-Thinking and Doubao generated relatively shorter stems, at 30.50 (23.25, 40.75) and 27.00 (23.00, 35.75) characters, respectively (Table 2). Pairwise comparisons showed that ChatGPT-5.4 generated significantly longer stems than Gemini 3 (Bonferroni-adjusted *p* = 0.014), whereas no statistically significant differences were observed between ChatGPT-5.4 and DeepSeek-V3.1, between Gemini 3 and DeepSeek-V3.1, or between Qwen3-Max-Thinking and Doubao. ChatGPT-5.4, Gemini 3, and DeepSeek-V3.1 each generated significantly longer stems than Qwen3-Max-Thinking and Doubao (all adjusted *p* < 0.001; Figure 2a; Supplementary Table S1).

For mean option length, ChatGPT-5.4 generated the longest options, at 16.89 ± 4.41 characters, followed by Gemini 3 at 14.62 ± 5.19 characters. Doubao generated options of intermediate length at 12.43 ± 3.86 characters, whereas DeepSeek-V3.1 and Qwen3-Max-Thinking generated relatively shorter options, at 10.25 (7.56, 12.69) and 9.00 (5.25, 10.25) characters, respectively (Table 2). Pairwise comparisons showed that all model pairs differed significantly after Bonferroni correction (adjusted *p* ≤ 0.047; Figure 2b; Supplementary Table S2).

For explanation length, Gemini 3 and ChatGPT-5.4 generated the longest explanations, at 134.03 ± 11.93 and 128.00 (110.25, 137.00) characters, respectively. Doubao and Qwen3-Max-Thinking were intermediate, at 81.07 ± 4.24 and 75.58 ± 14.52 characters, respectively, whereas DeepSeek-V3.1 generated the shortest explanations, at 59.50 (48.00, 73.00) characters (Table 2). Pairwise comparisons showed no statistically significant differences between ChatGPT-5.4 and Gemini 3 or between Qwen3-Max-Thinking and Doubao after Bonferroni correction (both adjusted *p* = 0.091). All other pairwise comparisons were statistically significant (all adjusted *p* < 0.001; Figure 2c; Supplementary Table S3).

The pattern for total response length largely mirrored that for explanation length. ChatGPT-5.4 and Gemini 3 generated the longest overall outputs, at 245.98 ± 39.61 and 239.67 ± 29.12 characters, respectively. DeepSeek-V3.1, Qwen3-Max-Thinking, and Doubao generated relatively shorter overall outputs, at 153.50 (129.00, 183.75), 143.00 (126.00, 160.25), and 162.80 ± 18.35 characters, respectively (Table 2). Pairwise comparisons showed no statistically significant difference between ChatGPT-5.4 and Gemini 3 or between DeepSeek-V3.1 and Doubao after Bonferroni correction. All remaining pairwise comparisons were statistically significant, including DeepSeek-V3.1 versus Qwen3-Max-Thinking (adjusted *p* = 0.001) and Qwen3-Max-Thinking versus Doubao (adjusted *p* < 0.001; Figure 2d; Supplementary Table S4).

For option length SD, Qwen3-Max-Thinking had the lowest value, at 1.54 (1.30, 2.54), indicating the most balanced option-length distribution. The other four models showed relatively greater dispersion, with values of 3.44 (2.33, 5.82) for ChatGPT-5.4, 4.23 (2.41, 5.85) for Gemini 3, 3.90 (1.57, 4.82) for DeepSeek-V3.1, and 4.23 ± 2.20 for Doubao (Table 2). Pairwise comparisons showed that Qwen3-Max-Thinking had significantly lower option-length imbalance than each of the other four models (all adjusted *p* < 0.001), whereas no statistically significant differences were observed among the other four models (Figure 2e; Supplementary Table S5).

For generation efficiency, Gemini 3 was the fastest, with a mean response time of 6.47 ± 1.27 s. Doubao and ChatGPT-5.4 followed, at 9.45 ± 1.47 s and 9.54 ± 1.43 s, respectively. DeepSeek-V3.1 and Qwen3-Max-Thinking required longer times, at 16.58 ± 3.46 s and 21.99 ± 4.38 s, respectively (Table 2). Pairwise comparisons showed no statistically significant difference between ChatGPT-5.4 and Doubao after Bonferroni correction (adjusted *p* = 1.000), whereas all other comparisons were statistically significant (all adjusted *p* < 0.001; Figure 2f; Supplementary Table S6).

### 3.3 Expert subjective ratings and inter-rater agreement

**Figure 3:**
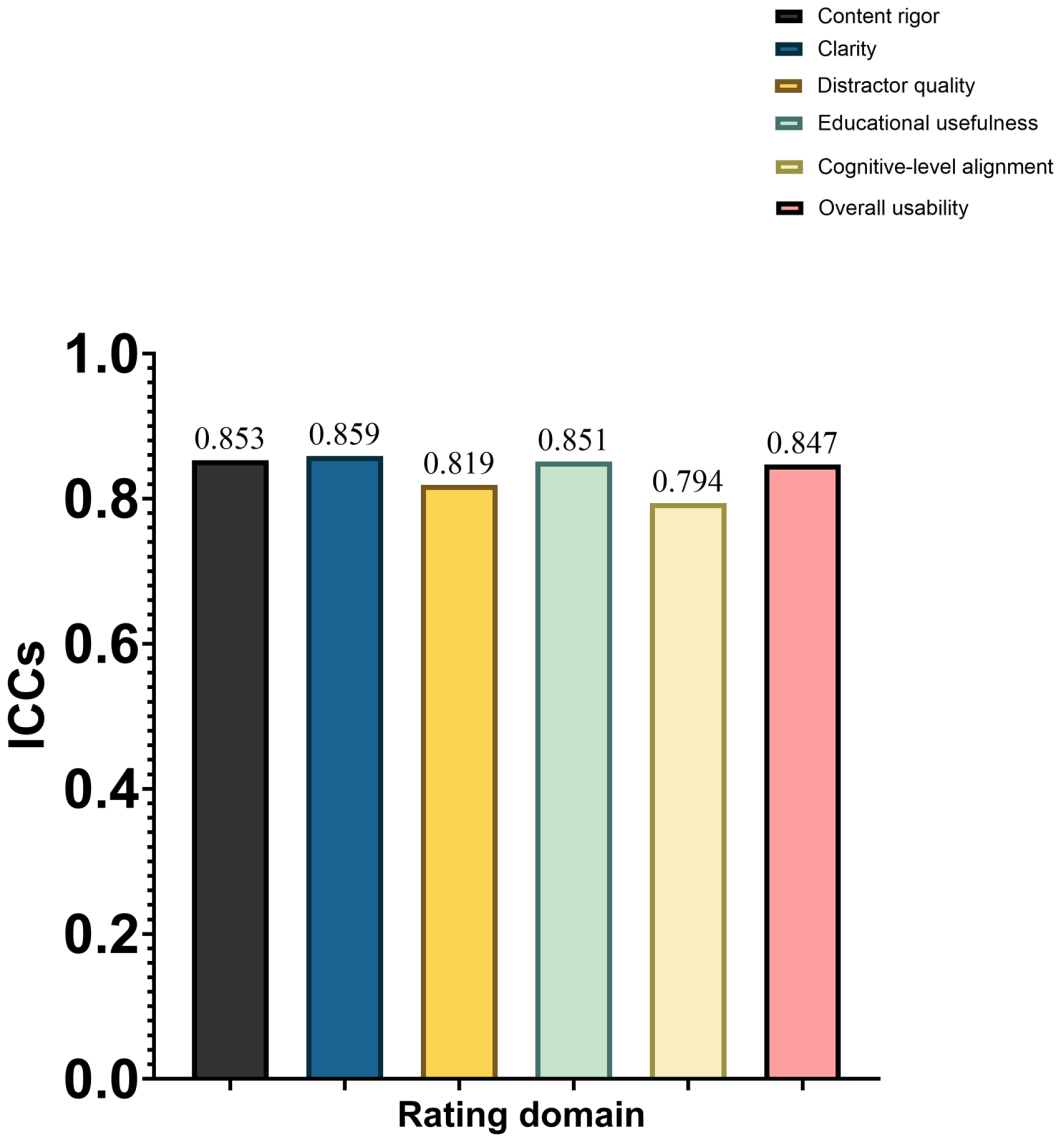
Inter-rater agreement across six expert-rated evaluation domains. Bar plots show the intraclass correlation coefficients (ICCs) for content rigor, clarity, distractor quality, educational usefulness, cognitive-level alignment, and overall usability, as independently rated by two retinal specialists. The ICC values are shown above the bars and indicate generally good inter-rater agreement across all subjective evaluation domains.

All subjective rating domains were non-normally distributed and are therefore presented as median (Q1, Q3), as shown in Table 3. Inter-rater agreement was generally good, with high ICC values across all domains. Specifically, the ICC was 0.853 (95% CI: 0.819–0.881) for content rigor, 0.859 (95% CI: 0.826–0.886) for clarity, 0.819 (95% CI: 0.778–0.853) for distractor quality, 0.851 (95% CI: 0.816–0.879) for educational usefulness, 0.794 (95% CI: 0.748–0.832) for cognitive-level alignment, and 0.847 (95% CI: 0.812–0.876) for overall usability (Figure 3).

**Table 3:** Comparison of expert subjective ratings of DR MCQs generated by five LLMs.

Rating domain	ChatGPT-5.4	Gemini 3	DeepSeek-V3.1	Qwen3-Max-Thinking	Doubao	ICC (95%CI)	Friedman χ^2^	Kendall’s W	*p*-values
Content rigor	5.00 (5.00, 5.00)	5.00 (5.00,5.00)	5.00 (5.00,5.00)	4.00 (4.00, 5.00)	5.00 (5.00,5.00)	0.853 (0.819, 0.881)	63.857	0.266	**<0.001**
Clarity	5.00 (4.00, 5.00)	5.00 (5.00,5.00)	4.00 (4.00,4.00)	5.00 (5.00, 5.00)	4.00 (4.00,4.00)	0.859 (0.826, 0.886)	155.860	0.649	**<0.001**
Distractor quality	4.00 (4.00, 4.00)	4.00 (4.00,4.00)	4.00 (4.00,4.00)	4.00 (4.00, 4.00)	3.00 (3.00,3.00)	0.819 (0.778, 0.853)	134.445	0.560	**<0.001**
Educational usefulness	5.00 (5.00, 5.00)	5.00 (5.00,5.00)	5.00 (5.00,5.00)	5.00 (5.00, 5.00)	5.00 (5.00,5.00)	0.851 (0.816, 0.879)	9.466	0.039	0.050
Cognitive-level alignment	4.00 (4.00, 4.00)	4.00 (4.00,4.00)	4.00 (4.00,4.00)	4.00 (4.00, 5.00)	4.00 (4.00,4.00)	0.794 (0.748, 0.832)	40.229	0.168	**<0.001**
Overall usability	5.00 (5.00, 5.00)	5.00 (5.00,5.00)	5.00 (5.00,5.00)	5.00 (5.00, 5.00)	5.00 (5.00,5.00)	0.847 (0.812, 0.876)	9.971	0.042	**0.041**
Mean score	4.67 (4.50, 4.67)	4.67 (4.67, 4.67)	4.50 (4.50, 4.50)	4.67 (4.67, 4.67)	4.33 (4.33, 4.33)	NA	101.311	0.422	**<0.001**

Data are presented as median (Q1, Q3). All rating domains were evaluated using a 5-point Likert scale. ICC values represent inter-rater agreement between the two independent expert raters and are presented with 95% CIs. The *p*-values shown in the table refer to overall between-model comparisons performed using the Friedman test, with task ID treated as the blocking factor. Kendall’s W was reported as the effect-size measure for the Friedman test. When the overall Friedman test was statistically significant, post hoc pairwise comparisons were performed using paired Wilcoxon signed-rank tests with Bonferroni correction; therefore, a statistically significant overall Friedman test does not necessarily indicate significant pairwise differences after correction. Bold *p*-values indicate statistically significant overall Friedman test results (*p* < 0.05). The three-category usability classification shown in Table 4 was derived from the 5-point overall usability score according to the prespecified conversion rule: score 5 = directly usable, score 4 = usable after minor revision, and scores 1–3 = not recommended.

LLM, large language model; MCQ, multiple-choice question; ICC, intraclass correlation coefficient; CI, confidence interval; Q1, first quartile; Q3, third quartile.

Overall comparisons using the Friedman test showed significant between-model differences in content rigor, clarity, distractor quality, cognitive-level alignment, overall usability, and the mean score across the six subjective rating domains. Educational usefulness did not reach statistical significance (*p* = 0.050) (Table 3). For overall usability, the Friedman test showed a weak but statistically significant overall difference across models (χ^2^ = 9.971, *p* = 0.041, Kendall’s W = 0.042), but no pairwise comparison remained statistically significant after Bonferroni correction.

**Figure 4:**
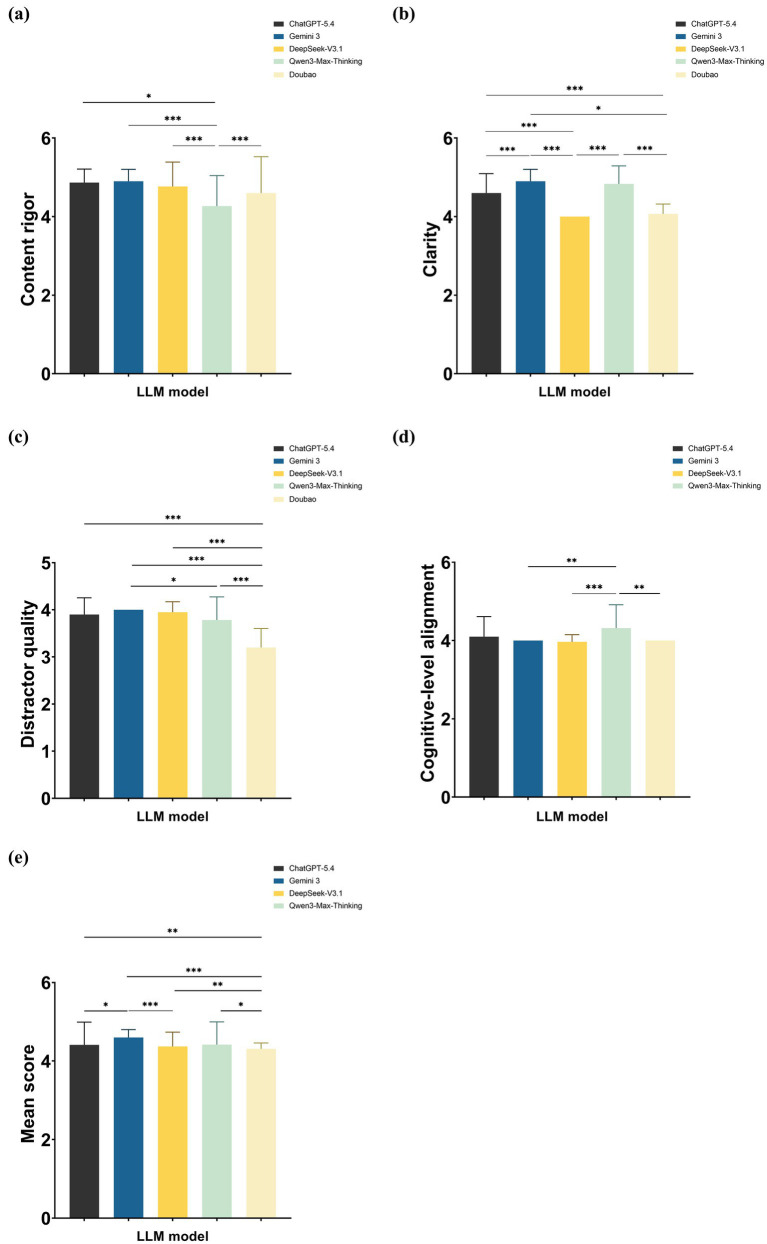
Comparison of expert subjective ratings across five large language models (LLMs). Bar plots summarize between-model differences in **(a)** content rigor, **(b)** clarity, **(c)** distractor quality, **(d)** cognitive-level alignment, and **(e)** mean score across the six expert-rated domains. Brackets denote significant pairwise differences after paired Wilcoxon signed-rank tests with Bonferroni correction. *p < 0.05; **p < 0.01; ***p < 0.001 after Bonferroni correction. Educational usefulness is not shown because the overall Friedman test did not reach statistical significance, and overall usability is not shown because no pairwise comparison remained significant after Bonferroni correction despite a weak overall Friedman test difference.

For content rigor, the median score was 5.00 for ChatGPT-5.4, Gemini 3, DeepSeek-V3.1, and Doubao, whereas Qwen3-Max-Thinking scored slightly lower at 4.00 (4.00, 5.00) (Table 3). Pairwise comparisons showed that Qwen3-Max-Thinking scored significantly lower than ChatGPT-5.4 (adjusted *p* = 0.037), Gemini 3, DeepSeek-V3.1, and Doubao (all adjusted *p* < 0.001), whereas no statistically significant differences were observed among the other four models (Figure 4a; Supplementary Table S7).

For clarity, Gemini 3 and Qwen3-Max-Thinking performed best, both with a median of 5.00 (5.00, 5.00). ChatGPT-5.4 scored 5.00 (4.00, 5.00), whereas DeepSeek-V3.1 and Doubao both scored 4.00 (4.00, 4.00) (Table 3). Pairwise comparisons showed significant differences between ChatGPT-5.4 and Gemini 3, DeepSeek-V3.1, Qwen3-Max-Thinking, and Doubao (adjusted *p* < 0.001, <0.001, 0.028, and <0.001, respectively). Gemini 3 did not differ significantly from Qwen3-Max-Thinking, and DeepSeek-V3.1 did not differ significantly from Doubao; all other pairwise comparisons were statistically significant after Bonferroni correction (Figure 4b; Supplementary Table S8).

For distractor quality, the median score was 4.00 (4.00, 4.00) for ChatGPT-5.4, Gemini 3, DeepSeek-V3.1, and Qwen3-Max-Thinking, whereas Doubao scored lowest at 3.00 (3.00, 3.00) (Table 3). Pairwise comparisons showed that Doubao scored significantly lower than each of the other four models (all adjusted *p* < 0.001). An additional significant difference was observed between Gemini 3 and Qwen3-Max-Thinking (adjusted *p* = 0.018), whereas the remaining comparisons were not statistically significant after correction (Figure 4c; Supplementary Table S9).

For cognitive-level alignment, the median score was 4.00 for all models, but the upper bound of the score distribution was higher for Qwen3-Max-Thinking, at 4.00 (4.00, 5.00) (Table 3). Pairwise comparisons showed that Qwen3-Max-Thinking differed significantly from Gemini 3 (adjusted *p* = 0.003), DeepSeek-V3.1 (adjusted *p* < 0.001), and Doubao (adjusted *p* = 0.003), but did not differ significantly from ChatGPT-5.4 (adjusted *p* = 0.093). No statistically significant differences were observed among the remaining models (Figure 4d; Supplementary Table S10).

Educational usefulness remained high across all five models, with a median score of 5.00 for each model, and the overall Friedman test did not reach statistical significance (*p* = 0.050) (Table 3). Overall usability also had a median score of 5.00 for each model. Although the overall Friedman test for overall usability was statistically significant, the effect size was small (Kendall’s W = 0.042), and no pairwise comparison remained significant after Bonferroni correction.

When averaged across the six subjective rating domains, Gemini 3 and Qwen3-Max-Thinking achieved the highest median scores, both at 4.67 (4.67, 4.67). ChatGPT-5.4 ranked next at 4.67 (4.50, 4.67), followed by DeepSeek-V3.1 at 4.50 (4.50, 4.50), whereas Doubao scored lowest at 4.33 (4.33, 4.33) (Table 3). Pairwise comparisons showed that Gemini 3 scored significantly higher than ChatGPT-5.4 (adjusted *p* = 0.013), DeepSeek-V3.1 (adjusted *p* < 0.001), and Doubao (adjusted *p* < 0.001), but did not differ significantly from Qwen3-Max-Thinking. ChatGPT-5.4 scored significantly higher than Doubao (adjusted *p* = 0.003), but did not differ significantly from DeepSeek-V3.1 or Qwen3-Max-Thinking. DeepSeek-V3.1 scored significantly higher than Doubao (adjusted *p* = 0.006), and Qwen3-Max-Thinking also scored significantly higher than Doubao (adjusted *p* = 0.015). The difference between DeepSeek-V3.1 and Qwen3-Max-Thinking did not remain statistically significant after Bonferroni correction (adjusted *p* = 0.052) (Figure 4e; Supplementary Table S11).

### 3.4 Supplementary classification results for overall usability

The supplementary classification results for overall usability are presented in Table 4. Gemini 3 had the highest proportion of items classified as directly usable, at 54/60 (90.00%; 95% CI: 79.85–95.34%), followed by ChatGPT-5.4 at 52/60 (86.67%; 95% CI: 75.83–93.09%) and DeepSeek-V3.1 at 50/60 (83.33%; 95% CI: 71.97–90.69%). Qwen3-Max-Thinking and Doubao each had 48/60 (80.00%; 95% CI: 68.22–88.17%) items classified as directly usable.

**Table 4:** Three-category classification derived from the overall usability Likert score of DR MCQs generated by five LLMs.

Overall usability category	ChatGPT-5.4 (*n* = 60)	Gemini 3 (*n* = 60)	DeepSeek-V3.1 (*n* = 60)	Qwen3-Max-Thinking (*n* = 60)	Doubao (*n* = 60)
Directly usable, *n* (%) [95% CI]	52 (86.67) [75.83, 93.09]	54 (90.00) [79.85, 95.34]	50 (83.33) [71.97, 90.69]	48 (80.00) [68.22, 88.17]	48 (80.00) [68.22, 88.17]
Usable after minor revision, *n* (%)	8 (13.33)	6 (10.00)	8 (13.33)	9 (15.00)	7 (11.67)
Not recommended, *n* (%)	0 (0.00)	0 (0.00)	2 (3.33)	3 (5.00)	5 (8.33)

This table presents the categorical distribution derived from the expert-rated 5-point overall usability Likert score. According to the prespecified conversion rule, an overall usability score of 5 was classified as directly usable, a score of 4 as usable after minor revision, and scores of 1–3 as not recommended without major revision. Data are presented as *n* (%). Wilson 95% CIs are provided for the directly usable category because it represents a key expert-rated usability proportion. This classification reflects expert judgment of item usability and should not be interpreted as evidence of psychometric validity based on learner testing.

CI, confidence interval; DR, diabetic retinopathy; LLM, large language model; MCQ, multiple-choice question.

For the category of usable after minor revision, the proportions were generally similar across models: 8/60 (13.33%) for ChatGPT-5.4, 6/60 (10.00%) for Gemini 3, 8/60 (13.33%) for DeepSeek-V3.1, 9/60 (15.00%) for Qwen3-Max-Thinking, and 7/60 (11.67%) for Doubao (Table 4).

No items were classified as not recommended for either ChatGPT-5.4 or Gemini 3. In contrast, the corresponding proportions were 2/60 (3.33%) for DeepSeek-V3.1, 3/60 (5.00%) for Qwen3-Max-Thinking, and 5/60 (8.33%) for Doubao (Table 4). Although the 5-point overall usability Likert score showed a weak but statistically significant overall difference in the Friedman test, no pairwise comparison remained significant after Bonferroni correction. Therefore, the three-category usability classification was interpreted descriptively. Overall, Gemini 3 and ChatGPT-5.4 yielded relatively higher proportions of directly usable items.

### 3.5 Correlations among textual output characteristics, generation efficiency, and expert-rated quality

**Figure 5:**
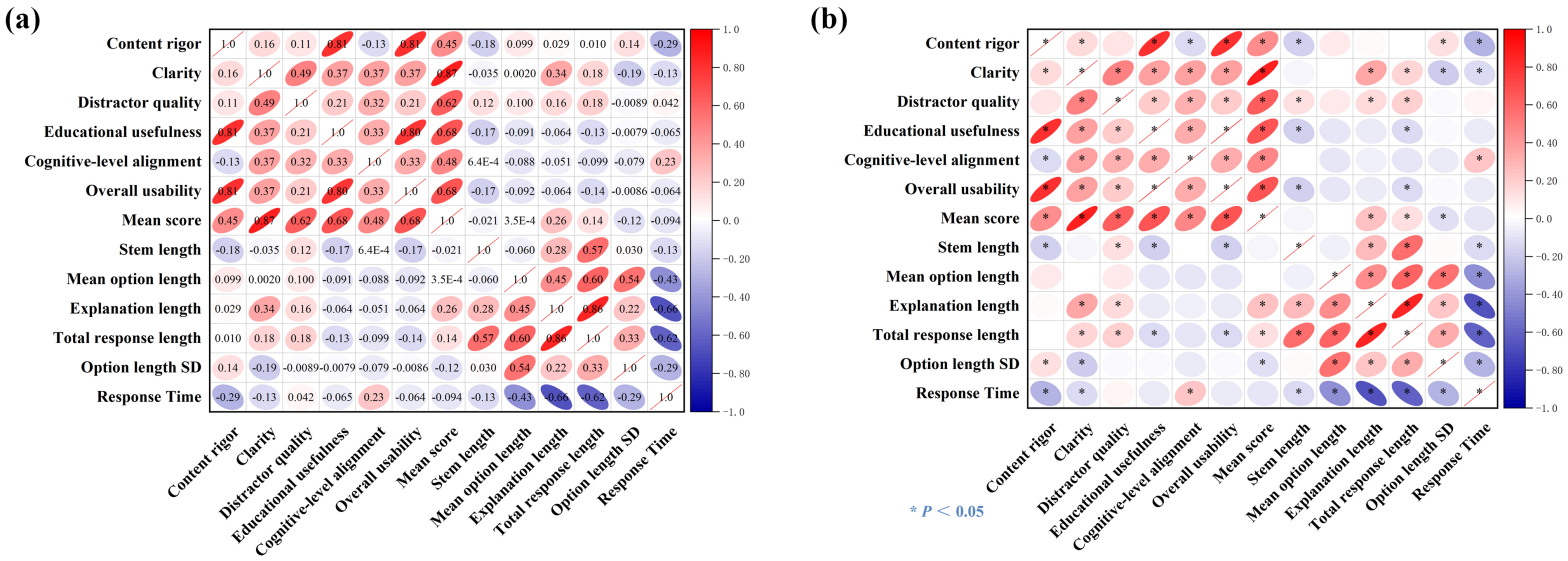
Spearman correlation analysis of expert-rated quality, textual output characteristics, and generation efficiency. **(a)** Correlation coefficient matrix showing the strength and direction of pairwise Spearman correlations among subjective rating domains, mean score, textual output characteristics, and response time. Numerical values within the cells represent correlation coefficients. Red indicates positive correlations, whereas blue indicates negative correlations. **(b)** Corresponding significance matrix for the same variables; asterisks indicate statistically significant correlations. Variables included content rigor, clarity, distractor quality, educational usefulness, cognitive-level alignment, overall usability, mean score, stem length, mean option length, explanation length, total response length, option length standard deviation (SD), and response time. Text-length variables are expressed as character counts, and response time is expressed in seconds. *p < 0.05.

Exploratory Spearman correlation analysis was performed to assess the relationships among textual output characteristics, generation efficiency, and expert subjective ratings, as shown in Figure 5. Overall, the subjective rating domains were predominantly positively correlated with one another. Among the strongest positive correlations, educational usefulness was strongly correlated with overall usability (*ρ* = 0.80, *p* < 0.05), while content rigor was strongly correlated with educational usefulness (*ρ* = 0.81, *p* < 0.05) and overall usability (*ρ* = 0.81, *p* < 0.05). Clarity was moderately positively correlated with distractor quality (*ρ* = 0.49, *p* < 0.05), cognitive-level alignment (*ρ* = 0.37, *p* < 0.05), educational usefulness (*ρ* = 0.37, *p* < 0.05), and overall usability (*ρ* = 0.37, *p* < 0.05). The mean score across the six subjective rating domains was positively correlated with each individual subjective domain, with the strongest correlation observed for clarity (*ρ* = 0.87, *p* < 0.05).

Regarding textual output characteristics, stem length showed weak negative correlations with content rigor (*ρ* = −0.18, *p* < 0.05), educational usefulness (*ρ* = −0.17, *p* < 0.05), and overall usability (*ρ* = −0.17, *p* < 0.05), indicating that longer stems were not associated with higher expert ratings. In contrast, explanation length was positively correlated with clarity (*ρ* = 0.34, *p* < 0.05), distractor quality (*ρ* = 0.16, *p* < 0.05), and the mean score across the six subjective rating domains (*ρ* = 0.26, *p* < 0.05), suggesting that more fully developed explanations were generally associated with better expert evaluation. Total response length was strongly positively correlated with explanation length (*ρ* = 0.86, *p* < 0.05) and moderately positively correlated with stem length (*ρ* = 0.57, *p* < 0.05) and mean option length (*ρ* = 0.60, *p* < 0.05), indicating that overall output length was mainly influenced by explanation length and option wording length.

Mean option length was positively correlated with explanation length (*ρ* = 0.45, *p* < 0.05), total response length (*ρ* = 0.60, *p* < 0.05), and option length SD (*ρ* = 0.54, *p* < 0.05), but showed no clear association with most subjective quality domains (all *p* ≥ 0.05). Option length SD was weakly positively correlated with content rigor (*ρ* = 0.14, *p* < 0.05), explanation length (*ρ* = 0.22, *p* < 0.05), and total response length (*ρ* = 0.33, *p* < 0.05), but weakly negatively correlated with clarity (*ρ* = −0.19, *p* < 0.05) and the mean score across the six subjective rating domains (*ρ* = −0.12, *p* < 0.05), suggesting that greater imbalance in option length may be associated with a slight decline in perceived overall item quality.

For generation efficiency, response time was negatively correlated with content rigor (*ρ* = −0.29, *p* < 0.05), stem length (*ρ* = −0.13, *p* < 0.05), mean option length (*ρ* = −0.43, *p* < 0.05), explanation length (*ρ* = −0.66, *p* < 0.05), total response length (*ρ* = −0.62, *p* < 0.05), and option length SD (*ρ* = −0.29, *p* < 0.05), but showed a weak positive correlation with cognitive-level alignment (*ρ* = 0.23, *p* < 0.05). Notably, response time did not show a positive relationship with overall quality indicators. Overall, these findings suggest that, under the public web-interface testing conditions of this study, longer response times did not translate into higher item quality. By contrast, more fully developed explanations showed some positive association with better clarity, distractor quality, and overall expert-rated mean score.

## 4 Discussion

This study systematically compared the performance of five publicly accessible LLMs in generating DR educational single-best-answer MCQs. The results showed that all five models were able to stably generate structurally complete and format-compliant MCQs, indicating that current mainstream LLMs have already acquired a relatively strong preliminary capacity for standardized item generation. However, clear differences remained across models in correct answer accuracy, textual output style, generation efficiency, distractor quality, cognitive-level alignment, and the proportion of directly usable items. Overall, ChatGPT-5.4 and Gemini 3 performed best in objective accuracy, Gemini 3 showed the most favorable overall balance in expert-rated usability, and ChatGPT-5.4, Qwen3-Max-Thinking, DeepSeek-V3.1, and Doubao each exhibited distinct strengths and limitations. Correlation analysis further suggested that longer stems did not necessarily correspond to higher item quality, whereas more fully developed explanations showed some positive association with better clarity and overall educational value. These findings suggest that the potential role of LLMs in medical education is no longer limited to “answering questions,” but is gradually expanding toward more application-oriented scenarios, including “generating educational content” and “assisting item design.” Previous studies have generally suggested that LLMs hold substantial promise in medicine and medical education (25), while also carrying risks related to content inaccuracy, limited transparency, and unstable reliability. Therefore, any high-stakes educational or clinical application should be built on rigorous validation and human oversight (26, 27). Our results are consistent with this overall view: although LLMs can already generate structurally acceptable DR item drafts with considerable stability, they are still not ready to enter formal question banks without review.

One of the most direct findings of this study was the very high structural stability observed across all five models: structurally qualified on first output, final structurally complete output, format compliance, and inclusion in subjective rating were all 100%. This suggests that, under a unified prompt framework and fixed output template, current mainstream LLMs are already able to follow complex instructions effectively and generate standardized MCQ outputs. From a methodological perspective, this result indicates that once task boundaries are clearly defined, output requirements are specific, and prompts are well designed, the ability of models to “generate items as instructed” is no longer the main obstacle. In other words, from the perspective of whether a model can produce questions at all, structured item generation is no longer the principal bottleneck. However, structural adequacy does not equate to content reliability. Although all models produced formally complete items, differences remained in correct answer accuracy, with ChatGPT-5.4 and Gemini 3 both reaching 100%, whereas the other models showed varying degrees of error. This distinction is particularly important in medical education, because for formal teaching and assessment, factual accuracy is far more critical than superficial linguistic fluency. Classical studies on medical MCQ construction have repeatedly emphasized that item-writing flaws and content errors not only undermine test validity but may also unfairly affect candidate performance (28, 29). Accordingly, structural standardization represents only the minimum threshold, whereas content accuracy is the core criterion determining whether an item can enter a formal question bank (30). In other words, the central challenge in current LLM-based automatic item generation has shifted from “whether items can be generated in the required format” to “whether medically correct items with a unique answer and explanation consistency can be generated reliably.” This also helps explain why, in clinical and medical education applications, LLMs are generally regarded as assistive tools rather than independent systems capable of replacing expert judgment (31).

Different models also showed distinct textual output styles, which represents another important finding of this study. ChatGPT-5.4 and DeepSeek-V3.1 generated longer stems, suggesting a greater tendency to provide more background information within the question stem. Gemini 3 and ChatGPT-5.4 produced longer explanations, indicating more fully developed explanatory output. Qwen3-Max-Thinking showed the lowest option length SD, suggesting a more balanced option-length distribution and a format more closely aligned with standardized MCQ design. These findings indicate that differences across models are reflected not only in overall quality, but also in item-writing style. Some models tended to favor information-rich stems and explanations, whereas others placed greater emphasis on balanced option structure and formal regularity. Such stylistic differences have practical educational relevance. High-quality MCQs in medical education should not simply pursue greater verbal richness, but rather achieve an appropriate balance between informational sufficiency and concise expression. Overly long stems, redundant cues, and non-parallel options may all compromise item quality; accordingly, longer output does not necessarily indicate better performance. Our correlation analysis supports this interpretation: stem length showed weak negative correlations with content rigor, educational usefulness, and overall usability, whereas explanation length was positively correlated with clarity and the mean score across the six subjective rating domains. In other words, more fully developed explanations may help enhance educational value, whereas longer stems do not necessarily improve item quality. These findings suggest that, in artificial intelligence-assisted item generation, improving output quality may depend less on simply increasing text length than on optimizing information organization and the relevance of explanations.

In terms of generation efficiency, Gemini 3 responded fastest, followed by ChatGPT-5.4 and Doubao, whereas DeepSeek-V3.1 and Qwen3-Max-Thinking required longer response times. Notably, longer response times did not translate into higher subjective quality ratings, suggesting that, in a public web-interface testing environment, slower generation does not necessarily mean better performance. This observation is consistent with findings from some previous ophthalmic LLM studies, which have shown that generation speed, linguistic verbosity, and practical educational usability do not always vary in parallel (32). It should be emphasized that response time in this study was closer to a user-experience-oriented efficiency metric than to a pure inference-latency metric. Its interpretation should therefore be cautious and should not be directly equated with the complexity of the model’s underlying reasoning process.

The expert ratings further revealed meaningful differences among models at the level of expert-rated instructional usability. For content rigor, all models remained at a generally high level except for Qwen3-Max-Thinking, which scored slightly lower. For clarity, Gemini 3 and Qwen3-Max-Thinking performed better. For distractor quality, Doubao was relatively weaker. Although all five models received generally high Likert ratings for educational usefulness and overall usability, the supplementary classification analysis showed that Gemini 3 and ChatGPT-5.4 yielded higher proportions of directly usable items. Educational usefulness did not show significant between-model differences, whereas Likert-scale overall usability showed only a weak overall difference and no significant pairwise differences after Bonferroni correction. This should not be interpreted as evidence that the models were equivalent in every educational respect. Rather, these findings suggest that all five models reached a generally high expert-rated usability threshold under the structured prompt and standardized DR MCQ blueprint. This may reflect the stabilizing effect of prespecified task domains, predefined knowledge points, controlled difficulty levels, cognitive-level requirements, and uniform output-format constraints. At the same time, educational usefulness and Likert-scale overall usability may be relatively less sensitive for differentiating model performance once generated items meet basic educational standards. These findings suggest that the differences among models are reflected more in the fine-grained quality of the generated items than in a simple binary distinction between “usable” and “unusable.” Unlike open-ended question answering, the core challenge in MCQ construction lies not merely in generating text that resembles a question, but in producing an item with a clearly defined single best answer, plausible distractors, and overall alignment with the intended educational objectives. Previous studies have noted that although LLM-generated medical MCQs are often satisfactory in formal completeness, limitations remain in distractor design, answer uniqueness, and the proportion of items that can be used directly in examinations without modification (33). Recent studies in ophthalmic education have likewise suggested that LLMs can generate ophthalmic questions with a certain degree of usability, but that formal incorporation into teaching or assessment still requires human review (34). Our findings are highly consistent with these reports, particularly in showing that a considerable proportion of items were judged directly usable by experts, while still supporting a practical workflow in which LLMs generate initial drafts that are subsequently reviewed and revised by experts. Particularly noteworthy is that Doubao performed relatively weakly in distractor quality and had the highest proportion of items classified as not recommended. This suggests that, at the current stage, distractor design remains a key weakness in automatic item generation for some LLMs. In fact, distractor quality has long been regarded as one of the most difficult components of MCQ construction to automate, because ideal incorrect options must be sufficiently plausible while still being clearly inferior to the correct answer, rather than becoming “partially correct” or valid under boundary conditions. This issue is especially critical in medical subspecialty education, because ambiguous or semi-correct distractors not only reduce discriminative value but may also mislead learners into forming inaccurate clinical knowledge frameworks. Therefore, even when a model can reliably generate structurally complete items, quality control at the level of distractor design still requires oversight by domain experts.

The correlation analysis in this study further adds to our understanding of what kinds of LLM outputs are more likely to receive higher expert ratings. First, the subjective rating domains were generally positively correlated with one another, particularly content rigor, educational usefulness, and overall usability, suggesting that high-quality items do not excel in only a single domain, but instead tend to perform well across multiple domains simultaneously. In other words, items with greater content rigor also tend to be more suitable for educational use and more likely to be judged by experts as overall usable. Second, stem length showed weak negative correlations with some quality indicators, whereas explanation length was positively correlated with clarity and the mean score across the six subjective rating domains. This suggests that, in DR educational MCQ generation, what truly contributes to quality is not indiscriminately increasing stem length, but rather providing more sufficient and educationally meaningful explanations. Good item design in medical education emphasizes necessary information and concise expression, rather than the simple accumulation of background details; in this regard, our correlation findings are educationally interpretable. At the same time, mean option length was positively correlated with total response length and explanation length, but showed no clear association with most subjective domains, indicating that longer options do not automatically translate into better distractor design. Finally, response time was negatively correlated with several text-length metrics and some quality indicators, further indicating that under the conditions of this study, longer web-interface response times did not confer a quality advantage. This finding has practical implications: in teaching support or question-bank drafting workflows, model selection should not be based solely on response speed, nor should one simply assume that “slower reasoning” necessarily corresponds to “higher item-generation quality.” It should be emphasized that this part of the analysis was exploratory and intended only to describe potential relationships among textual characteristics, generation efficiency, and item quality, and should not be interpreted causally.

Compared with previous studies, the present study shows clear continuity in two respects. First, prior research on the application of LLMs in medical education has generally concluded that, although LLMs demonstrate substantial potential in medicine and medical education, they are also affected by hallucinations, limited reproducibility, and the need for human review (35). The pattern observed in our DR educational MCQ generation task—namely, structural stability but continued need for content oversight—is a concrete manifestation of this broader pattern in the context of subspecialty item generation. Second, well-designed prompts and subsequent expert review are critical for improving item usability (36). After adopting a unified Chinese prompt template and a standardized item-generation blueprint, all models in our study achieved 100% structural completeness and format compliance, thereby providing empirical support for the view that high-quality prompts can substantially improve the formal quality of generated outputs. At the same time, this study also offers a degree of novelty. Most existing ophthalmic studies have primarily focused on model performance in answering pre-existing questions, whereas systematic comparisons of item-generation capability remain relatively limited (37–40). Even within the broader literature on automatic item generation, most studies have concentrated on general medical education or isolated course-specific settings (41–43). In contrast, the present study focused on DR, a highly clinically representative ophthalmic disease topic, and further organized the generation tasks into content domains including foundational knowledge, clinical cases, treatment decision-making, and screening/follow-up management, thereby making the evaluation more closely aligned with real-world subspecialty educational needs. In addition, this study did not assess “model quality” from a single perspective, but instead integrated objective evaluation, expert subjective ratings, inter-rater agreement analysis, and correlation analysis, thereby providing a more comprehensive picture of how different models perform in the context of ophthalmic subspecialty item generation. Recent studies have suggested that LLMs hold promise for ophthalmic educational question generation, but still require dedicated validation with a tighter thematic focus (21). The present study may be viewed as a further refinement and extension of that line of work.

From the perspective of educational application, our findings support positioning LLMs as assistive tools for ophthalmic question-bank construction and teaching preparation, rather than as independent systems capable of fully replacing expert item writers. For a high-frequency core topic such as DR, LLMs can rapidly generate structurally complete item drafts, a capability with practical value for expanding teaching cases, formative exercises, and residency training materials, especially in settings where faculty time is limited and question banks require rapid updating. At the same time, the present study also shows that directly incorporating unreviewed LLM outputs into formal examinations or other high-stakes educational assessments would still involve substantial risk. A more feasible approach, therefore, is not to pursue fully automated item generation, but rather to establish a human–machine collaborative workflow of “LLM draft generation–expert review and revision–standardized inclusion in the question bank.” From the broader perspective of digital health and digital public health tools, this also suggests that before LLM-based educational support systems are deployed in more formal and higher-risk application settings, they require clearly defined evaluation targets, appropriate classification frameworks, and quality-control strategies aligned with the needs of intended users (44, 45). The most realistic approach, and the one most consistent with current educational practice, is to use LLMs as draft-generation tools and to incorporate their outputs into standardized question-bank development through expert review, revision, and selection. Just as adherence to guidelines in clinical quality management can be evaluated using prespecified indicators, LLM-based item-generation systems should likewise establish quality evaluation and review mechanisms grounded in prespecified standards if they are to enter more standardized educational use (46). Within such a workflow, Gemini 3 and ChatGPT-5.4, given their higher correct answer accuracy and higher proportions of directly usable items, may be more suitable as first-stage drafting tools, whereas the other models may provide complementary value through different styles of stems, explanations, or cognitive-level design. In other words, the relationship among models may not be a simple hierarchy of superiority and inferiority, but rather one in which different models may assume different functional roles in future human–machine collaborative item-generation systems.

This study has several strengths. First, it focused on DR, a highly representative disease topic in ophthalmic education, rather than discussing ophthalmic questions in general, thereby increasing the specificity of the research question and its relevance to clinical education. DR itself encompasses key teaching content including disease definition, staging, DME, screening and follow-up, and treatment strategies, and is also highly consistent with mainstream guidelines and standardized training frameworks. Second, the use of a unified item-generation blueprint and a unified Chinese prompt template improved comparability across models to some extent. Third, the study integrated objective evaluation, expert subjective ratings, ICC-based agreement analysis, textual output characteristics, and exploratory correlation analysis, providing a relatively comprehensive assessment of model item-generation performance. Together, these design features strengthen the methodological completeness of the study and the interpretability of its findings in a subspecialty educational setting.

This study also has several limitations. First, it focused on a single disease topic, DR, and the findings therefore may not be directly generalizable to other ophthalmic subspecialties such as cataract, glaucoma, uveitis, or neuro-ophthalmology. Validation across a broader range of ophthalmic topics and knowledge-difficulty levels is still needed. Second, all models were tested through public web interfaces, and temperature, hidden system prompts, and some underlying parameters could not be fully standardized; thus, outputs may still have been influenced by platform-specific interface and system settings. Therefore, response time in this study should be interpreted as a user-perceived public-interface efficiency metric rather than a pure measure of model inference latency or reasoning complexity. Third, although agreement between the two expert raters was good, some subjective evaluation domains inevitably still involved expert judgment. Fourth, this study evaluated expert-rated item-generation quality, but did not administer the generated items to real learners; therefore, their actual difficulty, discrimination, and psychometric performance in examinations remain unknown. Fifth, because LLM versions evolve rapidly, the findings of this study are inherently time-sensitive, and future model iterations may alter the current comparative landscape. In addition, although the use of single-turn generation and a unified prompt strategy helped ensure comparability across models, it may also have underestimated the potential performance of some models under multi-turn interactive refinement. Future research could extend this work in several directions. First, the same framework could be applied to other ophthalmic disease topics to test the stability of LLM item-generation performance across different subspecialty scenarios. Second, by incorporating response data from real medical students or residents, future studies could perform psychometric validation of item difficulty, discrimination, and option functioning, thereby moving from “items judged good by experts” to “items proven effective in actual examinations.” Third, future work could compare question generation under different prompt languages, different prompt-engineering strategies, and even multimodal input conditions, with the aim of establishing a more mature human–machine collaborative workflow for ophthalmic item generation. Overall, these limitations do not weaken the main findings of the present study, but they do indicate that continued cross-topic, cross-platform, and cross-version validation will be necessary before LLM-based item generation can be extended to broader educational settings.

## 5 Conclusion

In summary, all five publicly accessible LLMs demonstrated promising potential for generating DR educational single-best-answer MCQs, although clear differences remained among models in content accuracy, clarity, distractor quality, direct usability, and public-interface efficiency. The findings support a cautious, expert-supervised use case in which LLMs generate first-draft items based on a standardized blueprint, while ophthalmology educators retain responsibility for verifying answer accuracy, guideline consistency, cognitive alignment, and distractor plausibility. With rigorous expert review and standardized workflows, LLM-assisted item generation may become a useful adjunct for ophthalmic education and question-bank development.

## Data Availability

The original contributions presented in the study are included in the article/Supplementary material, further inquiries can be directed to the corresponding authors.

## References

[1] Ikuno M. Concerning implications of large language models in medical education. Acad Med. (2026) 101:129. doi: 10.1093/acamed/wvaf023, 41617195

[2] Boscardin CK Abdulnour RE Gin BC. Macy foundation innovation report part I: current landscape of artificial intelligence in medical education. Acad Med. (2025) 100:S15–21. doi: 10.1097/ACM.0000000000006107, 40456178

[3] Shang Y Lin Y Li R Shang Y Li M Zhao L . The effectiveness of large language models in medical AI research for physicians: a randomized controlled trial. Cell Rep Med. (2025) 6:102469. doi: 10.1016/j.xcrm.2025.102469, 41308643 PMC12765838

[4] Xu Y Yang W. Editorial: artificial intelligence applications in chronic ocular diseases. Front Cell Dev Biol. (2023) 11:1295850. doi: 10.3389/fcell.2023.1295850, 38143924 PMC10740206

[5] Wu J Fang H Zhu J Zhang Y Li X Liu Y . Multi-rater prism: learning self-calibrated medical image segmentation from multiple raters. Sci Bull (Beijing). (2024) 69:2906–19. doi: 10.1016/j.scib.2024.06.037, 39155196

[6] Wan C Zhou X You Q Sun J Shen J Zhu S . Retinal image enhancement using cycle-constraint adversarial network. Front Med. (2022) 8:793726. doi: 10.3389/fmed.2021.793726, 35096883 PMC8789669

[7] Jiang L Jiang X Wu W Jiang F. Benchmarking publicly accessible large language models for high-myopia multiple-choice question generation in digital ophthalmic education and public health training. Front Public Health. (2026) 14:14 1843045. doi: 10.3389/fpubh.2026.1843045, 42163930 PMC13183845

[8] Jiang L Zhu Y Song C Hu X Fan X Yang W . Benchmarking large language models for congenital cataract parent counseling: safety, readability, and knowledge translation of developmental and genetic information. Front Cell Dev Biol. (2026) 14:1785731. doi: 10.3389/fcell.2026.1785731, 41970961 PMC13066122

[9] Cang X Ni M Song C Zhao J Guo Y Zou Y . ChatGPT-5 versus other mainstream large language models in core diabetic retinopathy patient queries. Front Cell Dev Biol. (2026) 14:1754221. doi: 10.3389/fcell.2026.1754221, 41960186 PMC13057549

[10] Chen SF Alyakin A Seas A Yang E Choi JJ Lee JV . LLM-assisted systematic review of large language models in clinical medicine. Nat Med. (2026) 32:1152–9. doi: 10.1038/s41591-026-04229-5, 41776077 PMC13004689

[11] Griot M Hemptinne C Vanderdonckt J Yuksel D. Large language models lack essential metacognition for reliable medical reasoning. Nat Commun. (2025) 16:642. doi: 10.1038/s41467-024-55628-6, 39809759 PMC11733150

[12] Kataoka Y So R. Benefits, limits, and risks of GPT-4 as an AI Chatbot for medicine. New Engl J Med. (2023) 388:2399–400. doi: 10.1056/NEJMc2305286, 37342940

[13] Jackson FI Keller NA Kouba I Kouba W Bracero LA Blitz MJ. Large language model clinical vignettes and multiple-choice questions for postgraduate medical education. Acad Med. (2025) 100:1163–6. doi: 10.1097/ACM.0000000000006137, 40550116

[14] Wu A Hao J Gao L Guo Y Hormel TT Flaxel CJ . Elevated retinal neovascularization on widefield optical coherence tomography angiography predicts complications in high-risk proliferative diabetic retinopathy. Am J Ophthalmol. (2025) 283:268–78. doi: 10.1016/j.ajo.2025.12.017, 41453592 PMC12834116

[15] Teo ZL Tham YC Yu M Chee ML Rim TH Cheung N . Global prevalence of diabetic retinopathy and projection of burden through 2045: systematic review and Meta-analysis. Ophthalmology. (2021) 128:1580–91. doi: 10.1016/j.ophtha.2021.04.027, 33940045

[16] Hou X Wang L Zhu D Guo L Weng J Zhang M . Prevalence of diabetic retinopathy and vision-threatening diabetic retinopathy in adults with diabetes in China. Nat Commun. (2023) 14:4296. doi: 10.1038/s41467-023-39864-w, 37463878 PMC10354077

[17] Cheung N Mitchell P Wong TY. Diabetic retinopathy. Lancet. (2010) 376:124–36. doi: 10.1016/S0140-6736(09)62124-3, 20580421

[18] Zhao J Lu Y Zhu S Li K Jiang Q Yang W. Systematic bibliometric and visualized analysis of research hotspots and trends on the application of artificial intelligence in ophthalmic disease diagnosis. Front Pharmacol. (2022) 13:13 930520. doi: 10.3389/fphar.2022.930520, 35754490 PMC9214201

[19] Wang R Zuo G Li K Li W Xuan Z Han Y . Systematic bibliometric and visualized analysis of research hotspots and trends on the application of artificial intelligence in diabetic retinopathy. Front Endocrinol. (2022) 13:13 1036426. doi: 10.3389/fendo.2022.1036426, 36387891 PMC9659570

[20] Gong D Fang L Cai Y Chong I Guo J Yan Z . Development and evaluation of a risk prediction model for diabetes mellitus type 2 patients with vision-threatening diabetic retinopathy. Front Endocrinol (Lausanne). (2023) 14:14 1244601. doi: 10.3389/fendo.2023.1244601, 37693352 PMC10484608

[21] Gholami S Mummert DB Wilson B Page S Dodhia R Lavista Ferres JM . Leveraging large language models to generate multiple-choice questions for ophthalmology education. Jama Ophthalmol. (2025) 143:955–61. doi: 10.1001/jamaophthalmol.2025.3622, 41100119 PMC12532029

[22] Wong TY Sun J Kawasaki R Ruamviboonsuk P Gupta N Lansingh VC . Guidelines on diabetic eye care. Ophthalmology. (2018) 125:1608–22. doi: 10.1016/j.ophtha.2018.04.007, 29776671

[23] Lim JI Kim SJ Bailey ST Kovach JL Vemulakonda GA Ying GS . Diabetic retinopathy preferred practice pattern®. Ophthalmology. (2025) 132:P75–P162. doi: 10.1016/j.ophtha.2024.12.020, 39918521

[24] American Diabetes Association Professional Practice Committee for Diabetes. 12 retinopathy, neuropathy, and foot care: standards of care in diabetes-2026. Diabetes Care. (2026) 49:S261–76. doi: 10.2337/dc26-S012 41358886 PMC12690177

[25] Thirunavukarasu AJ Ting DSJ Elangovan K Gutierrez L Tan TF Ting DSW. Large language models in medicine. Nat Med. (2023) 29:1930–40. doi: 10.1038/s41591-023-02448-8, 37460753

[26] Benítez TM Xu Y Boudreau JD Kow AWC Bello F Van Phuoc L . Harnessing the potential of large language models in medical education: promise and pitfalls. J Am Med Inform Assoc. (2024) 31:776–83. doi: 10.1093/jamia/ocad252, 38269644 PMC10873781

[27] Clusmann J Kolbinger FR Muti HS Carrero ZI Eckardt JN Laleh NG . The future landscape of large language models in medicine. Commun Med (Lond). (2023) 3:141. doi: 10.1038/s43856-023-00370-1, 37816837 PMC10564921

[28] Downing SM. The effects of violating standard item writing principles on tests and students: the consequences of using flawed test items on achievement examinations in medical education. Adv Health Sci Educ. (2005) 10:133–43. doi: 10.1007/s10459-004-4019-5, 16078098

[29] Tarrant M Ware J. Impact of item-writing flaws in multiple-choice questions on student achievement in high-stakes nursing assessments. Med Educ. (2008) 42:198–206. doi: 10.1111/j.1365-2923.2007.02957.x, 18230093

[30] Coughlin PA Featherstone CR. How to write a high quality multiple choice question (MCQ): a guide for clinicians. Eur J Vasc Endovasc. (2017) 54:654–8. doi: 10.1016/j.ejvs.2017.07.012, 28870436

[31] Lucas HC Upperman JS Robinson JR. A systematic review of large language models and their implications in medical education. Med Educ. (2024) 58:1276–85. doi: 10.1111/medu.15402, 38639098

[32] Srinivasan S Ai X Zou M Zou K Kim H Lo TWS . Ophthalmological question answering and reasoning using OpenAI o1 vs other large language models. JAMA Ophthalmol. (2025) 143:740–8. doi: 10.1001/jamaophthalmol.2025.2413, 40742581 PMC12314776

[33] Ch'en PY Day W Pekson RC Ch’en PY Barrientos J Burton WB . GPT-4 generated answer rationales to multiple choice assessment questions in undergraduate medical education. BMC Med Educ. (2025) 25:333. doi: 10.1186/s12909-025-06862-z, 40038669 PMC11877964

[34] Law AK So J Lui CT Choi YF Cheung KH Kei-ching Hung K . AI versus human-generated multiple-choice questions for medical education: a cohort study in a high-stakes examination. BMC Med Educ. (2025) 25:208. doi: 10.1186/s12909-025-06796-6, 39923067 PMC11806894

[35] Kameda Y Kaneko Y Ota K. Prompt validation in large language models for ophthalmology education. JAMA Ophthalmol. (2026) 144:366. doi: 10.1001/jamaophthalmol.2025.6397, 41746631

[36] Linde P Fichter F Dietlein M Sudbrock F Afshar K Dapper H . Psychometric properties and detectability of GPT-4o-generated multiple-choice questions compared with human-authored items across imaging specialties. NPJ Digit Med. (2026) 9:132. doi: 10.1038/s41746-025-02313-7, 41507355 PMC12881591

[37] Bedi S Liu Y Orr-Ewing L Dash D Koyejo S Callahan A . Testing and evaluation of health care applications of large language models: a systematic review. JAMA. (2025) 333:319–28. doi: 10.1001/jama.2024.21700, 39405325 PMC11480901

[38] Rocha H Chong YJ Thirunavukarasu AJ Wong YL Wong SW Chang YH . Performance of foundation models vs physicians in textual and multimodal ophthalmological questions. JAMA Ophthalmol. (2026) 144:5–13. doi: 10.1001/jamaophthalmol.2025.4255, 41231508 PMC12616532

[39] Cai LZ Shaheen A Jin A Fukui R Yi JS Yannuzzi N . Performance of generative large language models on ophthalmology board-style questions. Am J Ophthalmol. (2023) 254:141. doi: 10.1016/j.ajo.2023.05.024, 37339728

[40] Kleebayoon A Wiwanitkit V. Comment on: performance of generative large language models on ophthalmology board style questions. Am J Ophthalmol. (2023) 256:200. doi: 10.1016/j.ajo.2023.07.029, 37541409

[41] Safadi S Amirahmadi R Tlimat A Rovinski R Sun J Lee BW . Quality of human expert vs large language model-generated multiple-choice questions in the field of mechanical ventilation. Chest. (2025) 168:1425–32. doi: 10.1016/j.chest.2025.07.005, 40684906 PMC12795417

[42] Mistry NP Saeed H Rafique S le T Obaid H Adams SJ. Large language models as tools to generate radiology board-style multiple-choice questions. Acad Radiol. (2024) 31:3872–8. doi: 10.1016/j.acra.2024.06.046, 39013736

[43] Borowitz MJ Blackford AL Nagelia S Hruban RH. Large language models can generate high-quality pathology multiple-choice questions comparable with questions written by a human expert. Mod Pathol. (2025) 39:100940. doi: 10.1016/j.modpat.2025.100940, 41276086

[44] Maaß L Hrynyschyn R Lange M Löwe A Burdenski K Butten K . Challenges and alternatives to evaluation methods and regulation approaches for medical apps as Mobile medical devices: international and multidisciplinary focus group discussion. J Med Internet Res. (2024) 26:e54814. doi: 10.2196/54814, 39348678 PMC11474120

[45] Wienert J Jahnel T Maaß L. What are digital public health interventions? First steps toward a definition and an intervention classification framework. J Med Internet Res. (2022) 24:e31921. doi: 10.2196/31921, 35763320 PMC9277526

[46] Sacerdote C Bordon R Pitarella S Mano MP Baldi I Casella D . Compliance with clinical practice guidelines for breast cancer treatment: a population-based study of quality-of-care indicators in Italy. BMC Health Serv Res. (2013) 13:28. doi: 10.1186/1472-6963-13-28, 23351327 PMC3566978

