# Supplementary material for: Evaluating large language models for diabetic retinopathy multiple-choice question generation in clinical ophthalmic education
Source: Front Med (Lausanne). 2026 Jul 15;13:1874243. doi: 10.3389/fmed.2026.1874243 (PMC13416952; doi:10.3389/fmed.2026.1874243)
Supplement: Supplementary file 1 [file supplementary_file_1.docx]

**Supplementary Material 1**

**Diabetic Retinopathy (DR) Multiple-Choice Question Blueprint**

*(Numbered Separately by Four Content Domains)*

- Study objects: 5 large language models (LLMs)
- Question type: single-best-answer multiple-choice questions (single-best-answer MCQs)
- Content domains: foundational knowledge, clinical cases, treatment decision-making, screening/follow-up management
- Structure: 15 tasks per content domain, independently numbered within each category (Task 01-15)

**A. Foundational Knowledge (15 Tasks)**

*Header fields: Task No. | Knowledge Point | Difficulty | Cognitive Level | Item-Writing Instruction*

| **Task No.** | **Knowledge Point** | **Difficulty** | **Cognitive Level** | **Item-Writing Instruction** |
| --- | --- | --- | --- | --- |
| Task 01 | Basic definition of diabetic retinopathy | Easy | Recall | Create a standard conceptual question centered on the definition of DR. |
| Task 02 | Concept of non-proliferative diabetic retinopathy (NPDR) | Easy | Recall | Assess the basic definition or typical features of NPDR. |
| Task 03 | Concept of proliferative diabetic retinopathy (PDR) | Easy | Recall | Assess the definition of PDR and its core distinction from NPDR. |
| Task 04 | Definition of diabetic macular edema (DME) | Easy | Recall | Assess the concept of DME and its clinical significance. |
| Task 05 | Common fundus sign in DR: microaneurysms | Easy | Understanding | Identify the significance of microaneurysms in DR. |
| Task 06 | Common fundus sign in DR: dot-and-blot hemorrhages | Moderate | Understanding | Assess the basic association between hemorrhagic findings and DR staging. |
| Task 07 | Common fundus sign in DR: hard exudates | Moderate | Understanding | Assess the clinical significance of hard exudates and macular involvement. |
| Task 08 | Common fundus sign in DR: cotton-wool spots | Moderate | Understanding | Assess the ischemic significance of cotton-wool spots. |
| Task 09 | Clinical significance of neovascularization | Moderate | Understanding | Develop an item focused on the relationship between neovascularization and PDR. |
| Task 10 | Relationship between diabetes duration and DR risk | Moderate | Understanding | Assess the association between longer diabetes duration and the onset/progression of DR. |
| Task 11 | Glycemic control and risk of DR progression | Moderate | Application | Assess the basic principle linking HbA1c control with DR risk. |
| Task 12 | Relationship between hypertension and DR progression | Moderate | Application | Assess the effect of systemic risk factors on DR. |
| Task 13 | Basic principles of DR screening differences between type 1 and type 2 diabetes | Difficult | Analysis | Create an item requiring comparison of screening principles between the two patient groups. |
| Task 14 | Common mechanisms of vision loss caused by DR | Difficult | Analysis | Assess differentiation among mechanisms such as macular edema and vitreous hemorrhage. |
| Task 15 | Basic logic of DR severity stratification | Difficult | Analysis | Assess stratification thinking rather than rote memorization of grading criteria. |

**B. Clinical Cases (15 Tasks)**

*Header fields: Task No. | Knowledge Point | Difficulty | Cognitive Level | Item-Writing Instruction*

| **Task No.** | **Knowledge Point** | **Difficulty** | **Cognitive Level** | **Item-Writing Instruction** |
| --- | --- | --- | --- | --- |
| Task 01 | Recognition of early DR without obvious symptoms | Moderate | Application | Design a case involving a long diabetes history but no obvious chief complaint. |
| Task 02 | Clinical consideration of DME in a patient with blurred vision | Moderate | Application | Use symptoms and examination descriptions to elicit judgment of DME. |
| Task 03 | Fundus description suggesting mild NPDR | Moderate | Application | Provide a brief textual fundus description and ask for the most likely stage. |
| Task 04 | Fundus description suggesting moderate-to-severe NPDR | Moderate | Application | Assess recognition of more complex combinations of lesions. |
| Task 05 | Fundus description suggesting PDR | Moderate | Application | Use clues such as neovascularization or preretinal/vitreous hemorrhage to identify PDR. |
| Task 06 | Clinical reasoning when bilateral lesions are asymmetric | Difficult | Analysis | Assess judgment of differing degrees of involvement between the two eyes in a case. |
| Task 07 | Assessment of DR progression risk in a patient with diabetes and hypertension | Moderate | Application | Integrate systemic information to make a risk judgment. |
| Task 08 | Clinical judgment of DR in a patient with long-term poor glycemic control | Moderate | Application | Include elevated HbA1c as a clue in the case. |
| Task 09 | Considering vitreous hemorrhage in a patient with sudden vision loss | Difficult | Analysis | Construct an item using history and unavailable fundus visualization as clues. |
| Task 10 | Analysis of vision loss in a diabetic patient with mild fundus changes | Difficult | Analysis | Assess the ability to avoid judging solely from superficial fundus findings. |
| Task 11 | Identifying DME from textual OCT descriptions | Moderate | Application | Use textual descriptions only, without real images. |
| Task 12 | Judging ischemic or leakage features from textual FFA descriptions | Difficult | Analysis | Guide case judgment using text-based angiographic findings. |
| Task 13 | Clinical concerns regarding DR in pregnancy complicated by diabetes | Difficult | Analysis | Introduce a special population context and assess risk awareness. |
| Task 14 | Preoperative evaluation approach for a patient with cataract and DR | Difficult | Analysis | Assess judgment in the context of coexisting ocular disease. |
| Task 15 | Initial identification of DR in a primary-care first-visit setting | Moderate | Application | Set the case scenario in a primary-care or first-visit environment. |

**C. Treatment Decision-Making (15 Tasks)**

*Header fields: Task No. | Knowledge Point | Difficulty | Cognitive Level | Item-Writing Instruction*

| **Task No.** | **Knowledge Point** | **Difficulty** | **Cognitive Level** | **Item-Writing Instruction** |
| --- | --- | --- | --- | --- |
| Task 01 | General management principles for patients with mild NPDR | Moderate | Application | Develop an item around observation, metabolic control, and follow-up principles. |
| Task 02 | Next-step management approach for patients with moderate-to-severe NPDR | Moderate | Application | Assess decisions regarding specialist evaluation and further testing/follow-up. |
| Task 03 | Basic treatment direction for patients with PDR | Moderate | Application | Assess the principle that PDR requires active intervention. |
| Task 04 | Initial treatment approach for center-involving DME | Moderate | Application | Assess the preferred treatment direction. |
| Task 05 | Management principles for non-center-involving DME | Difficult | Analysis | Create an item requiring judgment based on visual acuity and lesion location. |
| Task 06 | Common indications for anti-VEGF therapy in DR/DME | Moderate | Understanding | Assess principles of anti-VEGF therapy. |
| Task 07 | Indications for panretinal photocoagulation (PRP) | Moderate | Understanding | Assess the correspondence between PRP and PDR. |
| Task 08 | Common indications for vitrectomy | Difficult | Analysis | Assess scenarios such as vitreous hemorrhage and tractional retinal detachment. |
| Task 09 | Management priorities when vitreous hemorrhage is present | Difficult | Analysis | Develop an item around acute vision loss and subsequent management. |
| Task 10 | Whether a patient with DR needs referral to a retinal specialist | Moderate | Application | Assess judgment of referral timing. |
| Task 11 | Role of systemic metabolic control in DR treatment | Moderate | Application | Integrate local ophthalmic treatment with systemic management. |
| Task 12 | Reasoning for patients with limited visual improvement after multiple treatments | Difficult | Analysis | Set a complex case emphasizing comprehensive judgment. |
| Task 13 | Considerations for DR treatment and surgical scheduling when cataract coexists | Difficult | Analysis | Assess reasoning about treatment sequence and risk evaluation. |
| Task 14 | Most appropriate next step for primary-care physicians facing suspected PDR | Moderate | Application | Emphasize initial management and timely referral. |
| Task 15 | Distinguishing "observation" from "immediate intervention" in treatment selection | Difficult | Analysis | Assess management boundaries across different severity levels. |

**D. Screening and Follow-up Management (15 Tasks)**

*Header fields: Task No. | Knowledge Point | Difficulty | Cognitive Level | Item-Writing Instruction*

| **Task No.** | **Knowledge Point** | **Difficulty** | **Cognitive Level** | **Item-Writing Instruction** |
| --- | --- | --- | --- | --- |
| Task 01 | Why patients with diabetes need regular fundus screening | Easy | Understanding | Assess the significance of screening and the concept of an asymptomatic stage. |
| Task 02 | Principles for initial fundus screening in patients with type 2 diabetes | Easy | Recall | Develop an item around the timing of initial screening. |
| Task 03 | Principles for initial fundus screening in patients with type 1 diabetes | Easy | Recall | Assess differences from type 2 diabetes. |
| Task 04 | Basic follow-up principles for patients without DR | Moderate | Application | Assess follow-up arrangements for patients without obvious lesions. |
| Task 05 | Follow-up principles for patients with mild NPDR | Moderate | Application | Assess follow-up logic in mild disease. |
| Task 06 | Follow-up management for patients with moderate-to-severe NPDR | Moderate | Application | Emphasize closer follow-up compared with mild NPDR. |
| Task 07 | Post-treatment follow-up management for patients with PDR | Difficult | Analysis | Assess awareness of monitoring after treatment. |
| Task 08 | Follow-up priorities for patients with DME | Moderate | Application | Assess tracking of visual acuity, macular status, and treatment response. |
| Task 09 | Fundus follow-up concerns in pregnancy complicated by diabetes | Difficult | Analysis | Assess screening and follow-up awareness for special populations. |
| Task 10 | Role of glycemic, blood pressure, and lipid management in long-term DR follow-up | Moderate | Application | Integrate ophthalmic follow-up with systemic management. |
| Task 11 | Patient education: follow-up is needed even without symptoms | Easy | Understanding | Suitable for developing an education-oriented MCQ. |
| Task 12 | Judging urgency of care-seeking in patients with sudden vision loss | Moderate | Application | Assess recognition of warning symptoms and timely re-examination. |
| Task 13 | Referral pathway after abnormalities are detected during primary-care screening | Moderate | Application | Develop an item around the management pathway after screening detects abnormalities. |
| Task 14 | Follow-up risk in patients with poor adherence | Difficult | Analysis | Assess adverse effects of long-term loss to follow-up on disease management. |
| Task 15 | Multidisciplinary collaboration in long-term DR management | Difficult | Analysis | Emphasize collaboration among ophthalmology, endocrinology, and patient self-management. |
