# Supplementary material for: Evaluating large language models for diabetic retinopathy multiple-choice question generation in clinical ophthalmic education
Source: Front Med (Lausanne). 2026 Jul 15;13:1874243. doi: 10.3389/fmed.2026.1874243 (PMC13416952; doi:10.3389/fmed.2026.1874243)
Supplement: Supplementary file 2 [file supplementary_file_2.docx]

**Supplementary Material 2**

**Exact Prompt Template and Standardized Remedial Prompt**

For formal testing, the placeholders in the main prompt template were filled according to the prespecified task blueprint for each item-generation task, including item category, specific knowledge point, difficulty level, and cognitive level. The standardized remedial prompt was used only when the initial output failed to meet the prespecified output format and was intended to correct formatting without changing the predefined task variables.

**A. 中文版**

**A1. 主提示词模板**

| 请你作为一名资深眼科教育命题专家，生成1道关于糖尿病视网膜病变的单项最佳选择题（single-best-answer multiple-choice question），面向眼科住院医师规范化培训学员。  命题要求如下： 1. 题目主题限定为糖尿病视网膜病变相关内容。 2. 题目类别为：【基础知识 / 临床病例 / 治疗决策 / 筛查与随访管理】中的一种。 3. 本题聚焦知识点：【填写具体知识点】。 4. 难度设定为：【简单 / 中等 / 困难】。 5. 认知层级设定为：【记忆 / 理解 / 应用 / 分析】。 6. 题型必须为单项最佳选择题，且仅有1个唯一最佳答案。 7. 请提供4个选项，标记为A、B、C、D。 8. 其余3个选项应具有一定干扰性，但必须为明确错误或明显次优选项，不能与正确答案重复、近义或在特定条件下同样成立。 9. 不要使用“以上都对”“以上都不对”等选项。 10. 不要设计依赖真实图片才能作答的题目；如需涉及检查结果，请使用文字描述。 11. 题干应简洁清晰、信息完整、逻辑自洽，避免歧义、双重否定、信息不足或过度提示。 12. 请确保题目内容、标准答案和解析符合当前主流眼科教育与临床实践常识，不得编造不存在的指南结论、检查阈值或治疗推荐。 13. 解析应简明、准确并具有教学性。 14. 除以下固定字段外，请勿添加其他说明。不要加入附加标题、前言、注释、考点标签或Markdown符号。  请严格按照以下格式输出：  题干： A. B. C. D. 正确答案： 解析： |
| --- |

**A2. 统一补救提示词**

| 请根据上一条指令，在不改变题目类别、具体知识点、难度设定和认知层级设定的前提下，重新生成并输出该题。请严格仅按照以下格式作答： 题干： A. B. C. D. 正确答案： 解析： 必须包含上述全部字段，不得添加任何额外说明，且仅允许1个唯一最佳答案。 |
| --- |

**B. English Version**

**B1. Main Prompt Template**

| Please act as a senior ophthalmic education item-writing expert and generate one single-best-answer multiple-choice question on Diabetic Retinopathy for ophthalmology residents in standardized residency training.  Requirements: 1. The topic must be restricted to diabetic retinopathy. 2. The item category must be one of the following: [basic knowledge / clinical case / treatment decision-making / screening and follow-up management]. 3. The question should focus on the following knowledge point: [insert specific knowledge point]. 4. Difficulty level: [easy / moderate / difficult]. 5. Cognitive level: [recall / comprehension / application / analysis]. 6. The item must be a single-best-answer multiple-choice question with only one unique best answer. 7. Please provide four options labeled A, B, C, and D. 8. The other three options should be plausibly distracting, but they must be clearly incorrect or clearly suboptimal, and must not duplicate the correct answer, be synonymous with it, or also become correct under specific conditions. 9. Do not use options such as “All of the above” or “None of the above.” 10. Do not design a question that depends on a real image for answering; if examination findings are needed, please describe them in words. 11. The stem should be concise, clear, sufficiently informative, and logically coherent, while avoiding ambiguity, double negatives, insufficient information, or excessive cueing. 12. Please ensure that the question content, correct answer, and explanation are consistent with current mainstream ophthalmic education and clinical practice, and do not fabricate non-existent guideline conclusions, examination thresholds, or treatment recommendations. 13. The explanation should be concise, accurate, and educational. 14. Do not add any content beyond the fixed fields below. Do not include any extra title, preface, note, topic tag, or Markdown symbols.  Please strictly use the following output format:  Stem: A. B. C. D. Correct answer: Explanation: |
| --- |

**B2. Standardized Remedial Prompt**

| Please follow the previous instruction and regenerate the item without changing the item category, specific knowledge point, difficulty level, or cognitive level. Please respond strictly in the following format only: Stem: A. B. C. D. Correct answer: Explanation: All of the above fields must be included. Do not add any extra commentary, and ensure that there is only one unique best answer. |
| --- |

**Note.** The remedial prompt was intended only to correct formatting when the initial output failed to meet the prespecified output structure; it was not intended to change the predefined task variables.
