# Supplementary material for: Evaluating large language models for diabetic retinopathy multiple-choice question generation in clinical ophthalmic education
Source: Front Med (Lausanne). 2026 Jul 15;13:1874243. doi: 10.3389/fmed.2026.1874243 (PMC13416952; doi:10.3389/fmed.2026.1874243)
Supplement: Supplementary file 3 [file supplementary_file_3.docx]

**Supplementary Material 3**

**Reference Standard and Evaluation Manual**

This appendix describes the reference standard and evaluation manual used to evaluate diabetic retinopathy (DR) multiple-choice questions (MCQs) generated by the five large language models (LLMs).

# 1. Purpose and scope

The purpose of the reference standard was to provide a consistent clinical and educational framework for judging correct-answer accuracy, content rigor, single-best-answer validity, and explanation consistency. The evaluation manual was used to standardize objective assessment, blinded expert scoring, categorical usability conversion, and discrepancy resolution.

The evaluation focused on single-best-answer MCQs generated for ophthalmology residents in standardized residency training. All generated items were evaluated in relation to the prespecified DR MCQ blueprint, which included 60 task units across four content domains: foundational knowledge, clinical cases, treatment decision-making, and screening/follow-up management. Each domain contained 15 task units, and each task specified a knowledge point, difficulty level, cognitive level, and item-writing instruction.

# 2. Development of the reference standard

The reference standard was developed before formal model-output evaluation by the research team based on mainstream ophthalmology textbooks, DR-related guidelines or consensus statements, and standardized ophthalmology residency-training materials. The reference standard was intended to reflect mainstream ophthalmic education and current clinical practice rather than a single institution-specific teaching preference.

Because the LLMs generated original items rather than answered a fixed question bank, the reference standard was not a fixed answer sheet. Instead, it served as a structured clinical and educational benchmark against which each generated stem, option set, keyed answer, and explanation were judged.

# 3. Knowledge scope covered by the reference standard

The reference standard covered the major DR-related knowledge areas represented in the prespecified blueprint:

1. Definition and general concepts of diabetic retinopathy;
2. Non-proliferative diabetic retinopathy (NPDR) and proliferative diabetic retinopathy (PDR);
3. Diabetic macular edema (DME) and its clinical relevance;
4. Common fundus signs, including microaneurysms, retinal hemorrhages, hard exudates, cotton-wool spots, and neovascularization;
5. Systemic risk factors, including diabetes duration, glycemic control, hypertension, and other metabolic factors;
6. Screening principles for patients with type 1 and type 2 diabetes;
7. Follow-up principles for patients with no DR, different severities of DR, and DME;
8. Treatment principles, including observation, systemic risk-factor control, anti-vascular endothelial growth factor therapy, retinal laser photocoagulation, panretinal photocoagulation, vitrectomy, and referral to a retinal specialist;
9. Special clinical scenarios, including pregnancy with diabetes, cataract with DR, vitreous hemorrhage, and primary-care or screening settings.

# 4. Expert review and finalization

The initial reference standard was drafted by retina-trained ophthalmologists and reviewed within the research team before formal scoring. The review focused on whether the standard was consistent with mainstream ophthalmic education, DR-related guideline or consensus principles, current clinical practice, and the intended cognitive level of each blueprint task. Any uncertainties were discussed by the research team before the final evaluation procedure. The finalized reference standard was then used together with the evaluation manual during blinded expert assessment.

# 5. Use of the reference standard during evaluation

| **Judgment domain** | **How the reference standard was applied** |
| --- | --- |
| Correct-answer accuracy | The model-indicated answer was judged as correct only if it was medically accurate, uniquely defensible as the best answer, and consistent with the information provided in the stem and options. |
| Single-best-answer validity | An item was considered valid only if one option was clearly the best answer. Items with more than one defensible correct answer, no clearly correct answer, or an answer that depended on unstated assumptions were considered problematic. |
| Content rigor | The item content, option design, and explanation were assessed for consistency with mainstream DR knowledge, ophthalmic education, and clinical practice. |
| Explanation consistency | The explanation was evaluated to determine whether it supported the keyed answer, avoided contradiction with the correct option, and provided a medically reasonable rationale. |
| Cognitive-level alignment | The generated item was compared with the prespecified cognitive level in the blueprint. Recall-level tasks were expected to test factual knowledge, whereas application- or analysis-level tasks were expected to require clinical interpretation, decision-making, or comparative reasoning. |
| Educational suitability | The item was judged for appropriateness for ophthalmology residents and for its potential use in teaching, formative assessment, or question-bank development. |

# 6. Objective evaluation criteria

| **Criterion** | **Definition and judgment rule** |
| --- | --- |
| Structural completeness | An item was considered structurally complete if it included all required components: a stem, four options labeled A-D, one indicated correct answer, and an explanation. |
| Format compliance | An item was considered format-compliant if it followed the single-best-answer MCQ format, included exactly four options, avoided multiple-answer structures, and did not use prohibited options such as “All of the above” or “None of the above.” |
| Correct-answer accuracy | Correct-answer accuracy was judged according to the reference standard. An item was classified as inaccurate if the keyed answer was medically incorrect, if more than one option could reasonably be defended as correct, if the explanation contradicted the keyed answer, or if the explanation lacked an acceptable medical rationale. |
| Textual output characteristics | Character count was used to quantify stem length, explanation length, total response length, mean option length, and option length standard deviation. Option length standard deviation was used to reflect imbalance in option length. |
| Generation efficiency | Response time was defined as the interval from prompt submission to completion of the visible model output in the public web interface. This metric was interpreted as user-perceived generation efficiency rather than pure model inference latency. |

# 7. Expert subjective evaluation domains and scoring anchors

Two ophthalmology experts independently rated each eligible item under anonymized and blinded conditions. Each item was evaluated on a 5-point Likert scale across six domains. Higher scores indicated better quality or stronger alignment with the intended educational purpose.

## 7.1 Content rigor

| **Score** | **Scoring anchor** |
| --- | --- |
| 5 | Completely accurate, rigorous, and consistent with mainstream DR education and clinical practice. |
| 4 | Generally accurate, with only minor wording or emphasis issues that do not affect correctness. |
| 3 | Partially acceptable, but with moderate limitations in precision, completeness, or clinical framing. |
| 2 | Contains important inaccuracies or omissions that may affect educational reliability. |
| 1 | Clearly inaccurate, misleading, or inconsistent with accepted DR knowledge. |

## 7.2 Clarity

| **Score** | **Scoring anchor** |
| --- | --- |
| 5 | Very clear, concise, and logically coherent, with no meaningful ambiguity. |
| 4 | Mostly clear, with only minor wording issues. |
| 3 | Understandable but somewhat ambiguous, verbose, or insufficiently focused. |
| 2 | Poorly worded or logically confusing, making interpretation difficult. |
| 1 | Unclear or incoherent, preventing reliable interpretation. |

## 7.3 Distractor quality

| **Score** | **Scoring anchor** |
| --- | --- |
| 5 | Distractors are plausible, clinically relevant, and well differentiated from the correct answer. |
| 4 | Distractors are generally reasonable, with minor weaknesses in plausibility or balance. |
| 3 | Some distractors are useful, but others are too obvious, weak, or uneven. |
| 2 | Distractors are largely implausible, poorly balanced, or potentially confusing. |
| 1 | Distractors are inappropriate, misleading, repetitive, or fail to support single-best-answer assessment. |

## 7.4 Educational usefulness

| **Score** | **Scoring anchor** |
| --- | --- |
| 5 | Highly suitable for educational use and clearly aligned with DR training needs. |
| 4 | Generally useful, requiring only minor refinement before educational use. |
| 3 | Moderately useful but requires noticeable revision to improve teaching value. |
| 2 | Limited educational value because of substantial content, clarity, or design problems. |
| 1 | Not educationally useful or unsuitable for ophthalmic training. |

## 7.5 Cognitive-level alignment

| **Score** | **Scoring anchor** |
| --- | --- |
| 5 | Fully aligned with the intended cognitive level. |
| 4 | Mostly aligned, with only minor deviation. |
| 3 | Partially aligned, but the required reasoning depth is not fully appropriate. |
| 2 | Poorly aligned with the intended cognitive level. |
| 1 | Clearly mismatched with the intended cognitive level. |

## 7.6 Overall usability

| **Score** | **Scoring anchor** |
| --- | --- |
| 5 | Directly usable; the item could be used for teaching or formative assessment after formatting checks only. |
| 4 | Usable after minor revision; the main content is correct, but limited wording, explanation, or distractor refinement is needed. |
| 3 | Not recommended without major revision; the item has moderate problems that require substantial revision before use. |
| 2 | Not recommended; the item has major problems affecting accuracy, clarity, distractor quality, or educational reliability. |
| 1 | Not recommended; the item is inaccurate, misleading, ambiguous, or unsuitable for educational use. |

# 8. Conversion of the overall usability score into three categories

For ease of result presentation and educational interpretation, the 5-point overall usability score was prespecified to be converted into three categories. These categories were directly derived from the Likert-scale overall usability score and were not assessed as an independent measure.

| **Category** | **Conversion rule** | **Interpretation** |
| --- | --- | --- |
| Directly usable | Overall usability score = 5 | The item could be used for teaching or formative assessment after formatting checks only. |
| Usable after minor revision | Overall usability score = 4 | The main content was correct, but limited wording, explanation, or distractor refinement was needed. |
| Not recommended | Overall usability score = 1-3 | The item required major revision or was unsuitable for educational use because of inaccuracy, ambiguity, weak distractors, cognitive-level mismatch, or explanation inconsistency. |

# 9. Discrepancy resolution

The two expert raters independently evaluated all eligible items. For Likert-scale ratings, inter-rater agreement was assessed using the intraclass correlation coefficient. For key judgments related to correct-answer accuracy, structural eligibility, content rigor, explanation consistency, and the overall usability classification, disagreements were reviewed against the reference standard and evaluation manual.

Disagreements were first resolved through discussion. If consensus could not be reached, a third senior retinal specialist was consulted for adjudication. For the three-category usability presentation, the final item-level category was determined according to the prespecified conversion rule from the overall usability score.

# 10. Relationship between the reference standard and the DR MCQ blueprint

The DR MCQ blueprint defined what each model was asked to generate, whereas the reference standard defined how the generated item was judged. The blueprint ensured that all models generated items for the same educational tasks, while the reference standard ensured that scoring was conducted using consistent clinical and educational criteria. Together, the blueprint, reference standard, and evaluation manual were used to improve fairness, reproducibility, and transparency in the comparison of LLM-generated DR MCQs.
