# Supplementary material for: Evaluating large language models for diabetic retinopathy multiple-choice question generation in clinical ophthalmic education
Source: Front Med (Lausanne). 2026 Jul 15;13:1874243. doi: 10.3389/fmed.2026.1874243 (PMC13416952; doi:10.3389/fmed.2026.1874243)
Supplement: Supplementary file 4 [file supplementary_file_4.docx]

**Supplementary Pairwise Comparison Matrices**

**Table S1. Pairwise Comparison Matrix for Stem Length Across the Five LLMs**

| **\|Z\| values \ P-values** | **ChatGPT-5.4** | **Gemini 3** | **DeepSeek-V3.1** | **Qwen3-Max-Thinking** | **Doubao** |
| --- | --- | --- | --- | --- | --- |
| **ChatGPT-5.4** | — | 0.014 | 1.000 | 0.000 | 0.000 |
| **Gemini 3** | 3.202 | — | 0.162 | 0.000 | 0.000 |
| **DeepSeek-V3.1** | 1.081 | 2.404 | — | 0.000 | 0.000 |
| **Qwen3-Max-Thinking** | 5.921 | 6.308 | 5.558 | — | 1.000 |
| **Doubao** | 6.168 | 6.554 | 5.556 | 1.596 | — |

***Note:***The lower triangle presents absolute |Z| values (standardized test statistics) from post hoc paired Wilcoxon signed-rank tests following the Friedman test, whereas the upper triangle presents Bonferroni-adjusted two-sided P-values. Values shown as 0.000 indicate P < 0.001. Diagonal dashes indicate within-model comparisons were not applicable.

**Table S2. Pairwise Comparison Matrix for Mean Option Length Across the Five LLMs**

| **\|Z\| values \ P-values** | **ChatGPT-5.4** | **Gemini 3** | **DeepSeek-V3.1** | **Qwen3-Max-Thinking** | **Doubao** |
| --- | --- | --- | --- | --- | --- |
| **ChatGPT-5.4** | — | 0.004 | 0.000 | 0.000 | 0.000 |
| **Gemini 3** | 3.556 | — | 0.000 | 0.000 | 0.000 |
| **DeepSeek-V3.1** | 6.380 | 5.254 | — | 0.002 | 0.047 |
| **Qwen3-Max-Thinking** | 6.515 | 5.994 | 3.707 | — | 0.000 |
| **Doubao** | 5.964 | 3.973 | 2.826 | 5.872 | — |

***Note:***The lower triangle presents absolute |Z| values (standardized test statistics) from post hoc paired Wilcoxon signed-rank tests following the Friedman test, whereas the upper triangle presents Bonferroni-adjusted two-sided P-values. Values shown as 0.000 indicate P < 0.001. Diagonal dashes indicate within-model comparisons were not applicable.

**Table S3. Pairwise Comparison Matrix for Explanation Length Across the Five LLMs**

| **\|Z\| values \ P-values** | **ChatGPT-5.4** | **Gemini 3** | **DeepSeek-V3.1** | **Qwen3-Max-Thinking** | **Doubao** |
| --- | --- | --- | --- | --- | --- |
| **ChatGPT-5.4** | — | 0.091 | 0.000 | 0.000 | 0.000 |
| **Gemini 3** | 2.608 | — | 0.000 | 0.000 | 0.000 |
| **DeepSeek-V3.1** | 6.722 | 6.722 | — | 0.000 | 0.000 |
| **Qwen3-Max-Thinking** | 6.670 | 6.737 | 4.190 | — | 0.091 |
| **Doubao** | 6.553 | 6.737 | 4.948 | 2.606 | — |

***Note:***The lower triangle presents absolute |Z| values (standardized test statistics) from post hoc paired Wilcoxon signed-rank tests following the Friedman test, whereas the upper triangle presents Bonferroni-adjusted two-sided P-values. Values shown as 0.000 indicate P < 0.001. Diagonal dashes indicate within-model comparisons were not applicable.

**Table S4. Pairwise Comparison Matrix for Total Response Length Across the Five LLMs**

| **\|Z\| values \ P-values** | **ChatGPT-5.4** | **Gemini 3** | **DeepSeek-V3.1** | **Qwen3-Max-Thinking** | **Doubao** |
| --- | --- | --- | --- | --- | --- |
| **ChatGPT-5.4** | — | 1.000 | 0.000 | 0.000 | 0.000 |
| **Gemini 3** | 1.005 | — | 0.000 | 0.000 | 0.000 |
| **DeepSeek-V3.1** | 6.622 | 6.585 | — | 0.001 | 1.000 |
| **Qwen3-Max-Thinking** | 6.722 | 6.736 | 3.858 | — | 0.000 |
| **Doubao** | 6.677 | 6.736 | 0.465 | 5.253 | — |

***Note:***The lower triangle presents absolute |Z| values (standardized test statistics) from post hoc paired Wilcoxon signed-rank tests following the Friedman test, whereas the upper triangle presents Bonferroni-adjusted two-sided P-values. Values shown as 0.000 indicate P < 0.001. Diagonal dashes indicate within-model comparisons were not applicable.

**Table S5. Pairwise Comparison Matrix for Option Length SD Across the Five LLMs**

| **\|Z\| values \ P-values** | **ChatGPT-5.4** | **Gemini 3** | **DeepSeek-V3.1** | **Qwen3-Max-Thinking** | **Doubao** |
| --- | --- | --- | --- | --- | --- |
| **ChatGPT-5.4** | — | 1.000 | 1.000 | 0.000 | 1.000 |
| **Gemini 3** | 0.928 | — | 1.000 | 0.000 | 1.000 |
| **DeepSeek-V3.1** | 1.428 | 1.568 | — | 0.000 | 1.000 |
| **Qwen3-Max-Thinking** | 4.604 | 5.338 | 4.491 | — | 0.000 |
| **Doubao** | 0.096 | 0.196 | 1.413 | 5.385 | — |

***Note:***The lower triangle presents absolute |Z| values (standardized test statistics) from post hoc paired Wilcoxon signed-rank tests following the Friedman test, whereas the upper triangle presents Bonferroni-adjusted two-sided P-values. Values shown as 0.000 indicate P < 0.001. Diagonal dashes indicate within-model comparisons were not applicable.

**Table S6. Pairwise Comparison Matrix for Response Times Across the Five LLMs**

| **\|Z\| values \ P-values** | **ChatGPT-5.4** | **Gemini 3** | **DeepSeek-V3.1** | **Qwen3-Max-Thinking** | **Doubao** |
| --- | --- | --- | --- | --- | --- |
| **ChatGPT-5.4** | — | 0.000 | 0.000 | 0.000 | 1.000 |
| **Gemini 3** | 6.478 | — | 0.000 | 0.000 | 0.000 |
| **DeepSeek-V3.1** | 6.692 | 6.736 | — | 0.000 | 0.000 |
| **Qwen3-Max-Thinking** | 6.736 | 6.736 | 5.286 | — | 0.000 |
| **Doubao** | 0.524 | 6.320 | 6.729 | 6.736 | — |

***Note:***The lower triangle presents absolute |Z| values (standardized test statistics) from post hoc paired Wilcoxon signed-rank tests following the Friedman test, whereas the upper triangle presents Bonferroni-adjusted two-sided P-values. Values shown as 0.000 indicate P < 0.001. Diagonal dashes indicate within-model comparisons were not applicable.

**Table S7. Pairwise Comparison Matrix for Content Rigor Across the Five LLMs**

| **\|Z\| values \ P-values** | **ChatGPT-5.4** | **Gemini 3** | **DeepSeek-V3.1** | **Qwen3-Max-Thinking** | **Doubao** |
| --- | --- | --- | --- | --- | --- |
| **ChatGPT-5.4** | — | 0.112 | 1.000 | 0.037 | 0.216 |
| **Gemini 3** | 2.537 | — | 1.000 | 0.000 | 1.000 |
| **DeepSeek-V3.1** | 1.472 | 1.613 | — | 0.000 | 1.000 |
| **Qwen3-Max-Thinking** | 2.899 | 4.956 | 4.413 | — | 0.000 |
| **Doubao** | 2.297 | 0.816 | 1.310 | 4.687 | — |

***Note:***The lower triangle presents absolute |Z| values (standardized test statistics) from post hoc paired Wilcoxon signed-rank tests following the Friedman test, whereas the upper triangle presents Bonferroni-adjusted two-sided P-values. Values shown as 0.000 indicate P < 0.001. Diagonal dashes indicate within-model comparisons were not applicable.

**Table S8. Pairwise Comparison Matrix for Clarity Across the Five LLMs**

| **\|Z\| values \ P-values** | **ChatGPT-5.4** | **Gemini 3** | **DeepSeek-V3.1** | **Qwen3-Max-Thinking** | **Doubao** |
| --- | --- | --- | --- | --- | --- |
| **ChatGPT-5.4** | — | 0.000 | 0.000 | 0.028 | 0.000 |
| **Gemini 3** | 4.025 | — | 0.000 | 1.000 | 0.000 |
| **DeepSeek-V3.1** | 6.000 | 7.348 | — | 0.000 | 0.455 |
| **Qwen3-Max-Thinking** | 2.985 | 1.027 | 6.804 | — | 0.000 |
| **Doubao** | 5.657 | 7.071 | 2.000 | 6.505 | — |

***Note:***The lower triangle presents absolute |Z| values (standardized test statistics) from post hoc paired Wilcoxon signed-rank tests following the Friedman test, whereas the upper triangle presents Bonferroni-adjusted two-sided P-values. Values shown as 0.000 indicate P < 0.001. Diagonal dashes indicate within-model comparisons were not applicable.

**Table S9. Pairwise Comparison Matrix for Distractor Quality Across the Five LLMs**

| **\|Z\| values \ P-values** | **ChatGPT-5.4** | **Gemini 3** | **DeepSeek-V3.1** | **Qwen3-Max-Thinking** | **Doubao** |
| --- | --- | --- | --- | --- | --- |
| **ChatGPT-5.4** | — | 0.339 | 1.000 | 0.196 | 0.000 |
| **Gemini 3** | 2.121 | — | 0.833 | 0.018 | 0.000 |
| **DeepSeek-V3.1** | 1.134 | 1.732 | — | 0.075 | 0.000 |
| **Qwen3-Max-Thinking** | 2.333 | 3.127 | 2.673 | — | 0.000 |
| **Doubao** | 6.062 | 6.928 | 6.564 | 5.105 | — |

***Note:***The lower triangle presents absolute |Z| values (standardized test statistics) from post hoc paired Wilcoxon signed-rank tests following the Friedman test, whereas the upper triangle presents Bonferroni-adjusted two-sided P-values. Values shown as 0.000 indicate P < 0.001. Diagonal dashes indicate within-model comparisons were not applicable.

**Table S10. Pairwise Comparison Matrix for Cognitive-level Alignment Across the Five LLMs**

| **\|Z\| values \ P-values** | **ChatGPT-5.4** | **Gemini 3** | **DeepSeek-V3.1** | **Qwen3-Max-Thinking** | **Doubao** |
| --- | --- | --- | --- | --- | --- |
| **ChatGPT-5.4** | — | 1.000 | 0.455 | 0.093 | 1.000 |
| **Gemini 3** | 1.500 | — | 1.000 | 0.003 | 1.000 |
| **DeepSeek-V3.1** | 2.000 | 1.414 | — | 0.000 | 1.000 |
| **Qwen3-Max-Thinking** | 2.600 | 3.657 | 3.900 | — | 0.003 |
| **Doubao** | 1.500 | 0.000 | 1.414 | 3.657 | — |

***Note:***The lower triangle presents absolute |Z| values (standardized test statistics) from post hoc paired Wilcoxon signed-rank tests following the Friedman test, whereas the upper triangle presents Bonferroni-adjusted two-sided P-values. Values shown as 0.000 indicate P < 0.001. Diagonal dashes indicate within-model comparisons were not applicable.

**Table S11. Pairwise Comparison Matrix for Mean Score Across the Five LLMs**

| **\|Z\| values \ P-values** | **ChatGPT-5.4** | **Gemini 3** | **DeepSeek-V3.1** | **Qwen3-Max-Thinking** | **Doubao** |
| --- | --- | --- | --- | --- | --- |
| **ChatGPT-5.4** | — | 0.013 | 0.235 | 1.000 | 0.003 |
| **Gemini 3** | 3.222 | — | 0.000 | 0.180 | 0.000 |
| **DeepSeek-V3.1** | 2.266 | 6.236 | — | 0.052 | 0.006 |
| **Qwen3-Max-Thinking** | 0.718 | 2.366 | 2.797 | — | 0.015 |
| **Doubao** | 3.590 | 6.850 | 3.438 | 3.174 | — |

***Note:***The lower triangle presents absolute |Z| values (standardized test statistics) from post hoc paired Wilcoxon signed-rank tests following the Friedman test, whereas the upper triangle presents Bonferroni-adjusted two-sided P-values. Values shown as 0.000 indicate P < 0.001. Diagonal dashes indicate within-model comparisons were not applicable.
